# Neuro293: A REST-knockout HEK-293 cell line enables the expression of neuron-restricted genes for the high-throughput testing of human neurobiology and the biochemistry of neuronal proteins

**DOI:** 10.1093/biomethods/bpaf036

**Published:** 2025-05-10

**Authors:** Joshua T Moses, Fahad B Shah, Nicholas M McVay, Dylan E Capes, Christopher C Bosse-Joseph, Jocelyn Salazar, Victoria K Slone, John E Eberth, Jonathan Satin, Andrew N Stewart

**Affiliations:** Department of Physiology, University of Kentucky, Lexington, KY 40536, United States; Spinal Cord and Brain Injury Research Center, University of Kentucky, Lexington, KY 40536, United States; College of Medicine, University of Kentucky, Lexington, KY 40536, United States; Spinal Cord and Brain Injury Research Center, University of Kentucky, Lexington, KY 40536, United States; College of Medicine, University of Kentucky, Lexington, KY 40536, United States; Department of Neuroscience, University of Kentucky, Lexington, KY 40536, United States; Department of Physiology, University of Kentucky, Lexington, KY 40536, United States; College of Medicine, University of Kentucky, Lexington, KY 40536, United States; Spinal Cord and Brain Injury Research Center, University of Kentucky, Lexington, KY 40536, United States; College of Medicine, University of Kentucky, Lexington, KY 40536, United States; Department of Neuroscience, University of Kentucky, Lexington, KY 40536, United States; Spinal Cord and Brain Injury Research Center, University of Kentucky, Lexington, KY 40536, United States; College of Medicine, University of Kentucky, Lexington, KY 40536, United States; Department of Neuroscience, University of Kentucky, Lexington, KY 40536, United States; Spinal Cord and Brain Injury Research Center, University of Kentucky, Lexington, KY 40536, United States; College of Medicine, University of Kentucky, Lexington, KY 40536, United States; Department of Neuroscience, University of Kentucky, Lexington, KY 40536, United States; Spinal Cord and Brain Injury Research Center, University of Kentucky, Lexington, KY 40536, United States; College of Medicine, University of Kentucky, Lexington, KY 40536, United States; Department of Neuroscience, University of Kentucky, Lexington, KY 40536, United States; Department of Physiology, University of Kentucky, Lexington, KY 40536, United States; College of Medicine, University of Kentucky, Lexington, KY 40536, United States; Department of Physiology, University of Kentucky, Lexington, KY 40536, United States; College of Medicine, University of Kentucky, Lexington, KY 40536, United States; Spinal Cord and Brain Injury Research Center, University of Kentucky, Lexington, KY 40536, United States; College of Medicine, University of Kentucky, Lexington, KY 40536, United States; Department of Neuroscience, University of Kentucky, Lexington, KY 40536, United States

**Keywords:** high-throughput testing, neuronal differentiation, mature neuronal proteins, stable cell line

## Abstract

Efficient interrogation of neurobiology remains bottlenecked by obtaining mature neurons. Immortalized cell lines still require lengthy differentiation periods to obtain neuron-like cells, which may not efficiently differentiate and are challenging to transfect with plasmids relative to other cell lines such as HEK-293’s. To overcome challenges with limited access to cells that express mature neuronal proteins, we knocked out the RE1-silencing transcription factor (REST) from HEK-293’s to create a novel neuron-like cell, which we name Neuro293. RNA-sequencing and bioinformatics analyses revealed a significant upregulation of genes associated with neurobiology and membrane excitability including pre-/post-synaptic proteins, voltage gated ion channels, neuron-cytoskeleton, as well as neurotransmitter synthesis, packaging, and release. Western blot validated the upregulation of Synapsin-1 (Syn1) and Snap-25 as two neuron-restricted proteins, as well as the potassium channel Kv1.2. Immunocytochemistry against Neurofilament 200 kd revealed a significant upregulation and accumulation in singular processes extending from Neuro293’s cell body. Similarly, while Syn1 increased in the cell body, Syn1 protein accumulated at the ends of processes extruding from Neuro293’s. Neuro293’s express reporter-genes through the Syn1 promoter after infection with adeno-associated viruses (AAV). However, transient transfection with AAV2 plasmids led to leaky expression through promoter-independent mechanisms. Despite an upregulation of many voltage-gated ion channels, Neuro293’s do not possess excitable membranes. Collectively, REST-knockout in HEK-293’s induces a quickly dividing and easily transfectable cell line that expresses neuron-restricted and mature neuronal proteins which can be used for high-throughput biochemical interrogation, however, without further modifications neither HEK-293’s or Neuro293’s exhibit properties of excitable membranes.

## Introduction

There is an expanding need to produce mature human neurons for *in vitro* experimentation. Differentiating neural progenitor cells or immortalized SH-SY5Y neuroblastoma cells is commonly used to obtain mature neurons of human origin [1, 2], while the use of primary neuronal cultures or administering similar differentiation strategies is available for rodents. In any situation, obtaining mature neurons and/or mature neuronal proteins is bottle-necked by time-consuming and often inefficient differentiation procedures [1, 3–5]. Obtaining mature neurons, particularly those of human origin, is vital for research progress, yet remains time-consuming and technically challenging to obtain.

The needs for neuron-like cell culture models are dependent upon the research question. For the interrogation of protein–protein interactions related to mature neuronal proteins, generating mature neurons is a requirement unless transient transfection methods are sufficient to simply generate the proteins of interest. Interrogating axon/dendrite growth or synaptic vesicle formation/trafficking also requires obtaining mature neurons through differentiation processes. However, investigating cellular excitability remains important for several non-neuroscience-related fields such as cardiology or muscular physiology. Excitable cells remain difficult to obtain without generating primary cultures and the differentiation of myocytes or cardiomyocytes. Previous attempts to generate a high-throughput line of excitable cells utilized the transfection of Kir2.1 into Human Embryonic Kidney Cells (HEK-293’s) to enable potassium channel currents to maintain the resting membrane potential below the action potential threshold [6, 7]. Kir2.1 expression on HEK-293’s remains an established method to interrogate membrane excitability and can be used in conjunction with transient transfection of ion channels of interest. There remains a need for a cell line that possesses the following characteristics: (i) an ability to express proteins through neuron-restricted promoters; (ii) exhibits high susceptibility to plasmid transfection and high protein expression; (iii) produces mature neuronal proteins for biochemical interrogation; (iv) has excitable membranes; (v) possess an ease of culture, expansion, and robust viability; and ideally (vi) display all of these characteristics without a need for long differentiation protocols. To address these issues, we knocked out the RE1-silencing transcription factor (REST-KO) from immortalized HEK-293’s to force the expression of mature neuronal proteins and evaluated membrane excitability through potassium currents. REST acts as a transcriptional repressor that governs the expression of many neuron-restricted proteins by binding to the Neuron-Restrictive Silencer Element (NSRE) found within the promoters of many mature neuronal proteins and is typically expressed in all cells except mature neurons [8–10].

Below, we present our characterization of this novel REST-KO HEK-293 cell line (named Neuro293). While Neuro293’s are a stable, expanding cell line that produce mature neuronal proteins, REST-KO alone remains insufficient to induce excitable membranes in HEK-293’s. We propose the use of REST-KO in HEK-293’s as a novel approach for the high-throughput interrogation of mature human neurobiology and biochemistry, for purposes outside of membrane excitability.

## Methods

### Cell culture and media conditions

HEK-293T’s were obtained from the American Type Culture Collection (ATCC) and maintained under cryopreservation as a routinely used cell line in the lab. Cells are maintained in culture conditions including Dulbecco’s Modified Eagle’s Medium (DMEM; ThermoFisher Scientific; 11965118) as a basal medium, including 10% Fetal Bovine Serum (FBS), and 1% Anti-Anti (ThermoFisher Scientific; 15240062; antibiotic-antimycotic at 100 U/ml penicillin, 100 µg/ml streptomycin, 0.25 µg/ml Amphotericin B). Cells are expanded to approximately 70%–80% confluence prior to passaging using 0.05% Trypsin (ThermoFisher Scientific; 15090046). Media conditions are not changed after REST-knockout. When performing immunocytochemistry (ICC) or electrophysiological experiments, 12-mm round cover slips were coated with Poly-d-Lysine and Laminin (Millipore Sigma; LPDL001) for adherence. Otherwise, cells are expanded as plastic adherent.

Doubling time was assessed by seeding cells at 1.0 × 10^4^, 2.5 × 10^4^, 5.0 × 10^4^, and 1.0 × 10^5^ in single wells of a 12-well plate and cultured for 72-h prior to harvesting and counting on a hemocytometer. Doubling time was calculated and averaged across all assessed wells for each group. The HEK-293 well seeded with 5.0 × 10^4^ cells exhibited significantly fewer cells than anticipated, giving total cell counts closer to that of 2.5 × 10^4^ seeding density and was not used for analysis due to concerns with pipetting error during seeding. All other wells from both groups gave comparable double time estimates.

### CRISPR knockout of REST

#### Plasmid production and design

CRISPR/Cas9 plasmids to generate REST-KO were synthesized at VectorBuilder. Further, guide arms were designed using the VectorBuilder guide-arm design tool. The complete expression plasmid includes the expression of both guide arms under a U6 promoter, the Cas9 protein under a CBh promoter, as well as an EGFP-PuroR under a cytomegalovirus (CMV) promoter (U6-Crispr guide arms, CBh-Cas9, CMV-EGFP/PuroR). Guide arm sequences were selected (1: CCGCAGCGGTCACAGCGAAT and 2: ATATGCGTACTCATTCAGGT) to flank the entirety of the prospective DNA binding domain of REST. Specifically, Cas9-targeted knockout was guided towards an ∼8569-bp fragment located between location 8409 and 16997 within the Homosapien REST gene on chromosome 4 (RefSeqGene: NG_029447.1; See Supplemental Materials 1 for the complete region sequence).

#### Transfection and antibiotic selection

When HEK-293 cells reached ∼70% confluence in a T25 cell culture flask, 5 µg of plasmid construct was used for transfection using Lipofectamine 3000 (ThermoFisher Scientific; L3000150; 5 µl P3000, 5 µl Lipofectamine). At 24-h post-transfection, green fluorescence was used to validate effective transfection and protein expression, and non-transfected cells were removed by adding 10 µg/ml Puromycin (Millipore Sigma; P4512) for 72-h into the culture. Dead cells were removed from the media and the surviving cells were allowed to expand until reaching 70% confluence again.

#### FACS sorting of Syn1^+^ cells

Once Crispr/Cas9-treated cells reached 70% confluence, cells were again transiently transfected using a plasmid that expresses mCherry under the synapsin-1 (Syn1) promoter as just described (pAAV-Syn1-mCherry; Addgene; Addgene ID: 114472). Cells expressing mCherry under the neuron-restricted Syn1 promoter were sorted using Fluorescence Activated Cell Sorting (FACS). Cells were collected after dissociation with Trypsin and resuspended in FACS buffer containing Hanks Buffered Saline Solution, 1% FBS, and 1% Anti-Anti. Cells were sorted by gaiting out debris and doublets and selecting for the highest expressing cells.

#### Clonal selection, genotyping, and off-target CRISPR/Cas9 analysis

**Figure 1.**
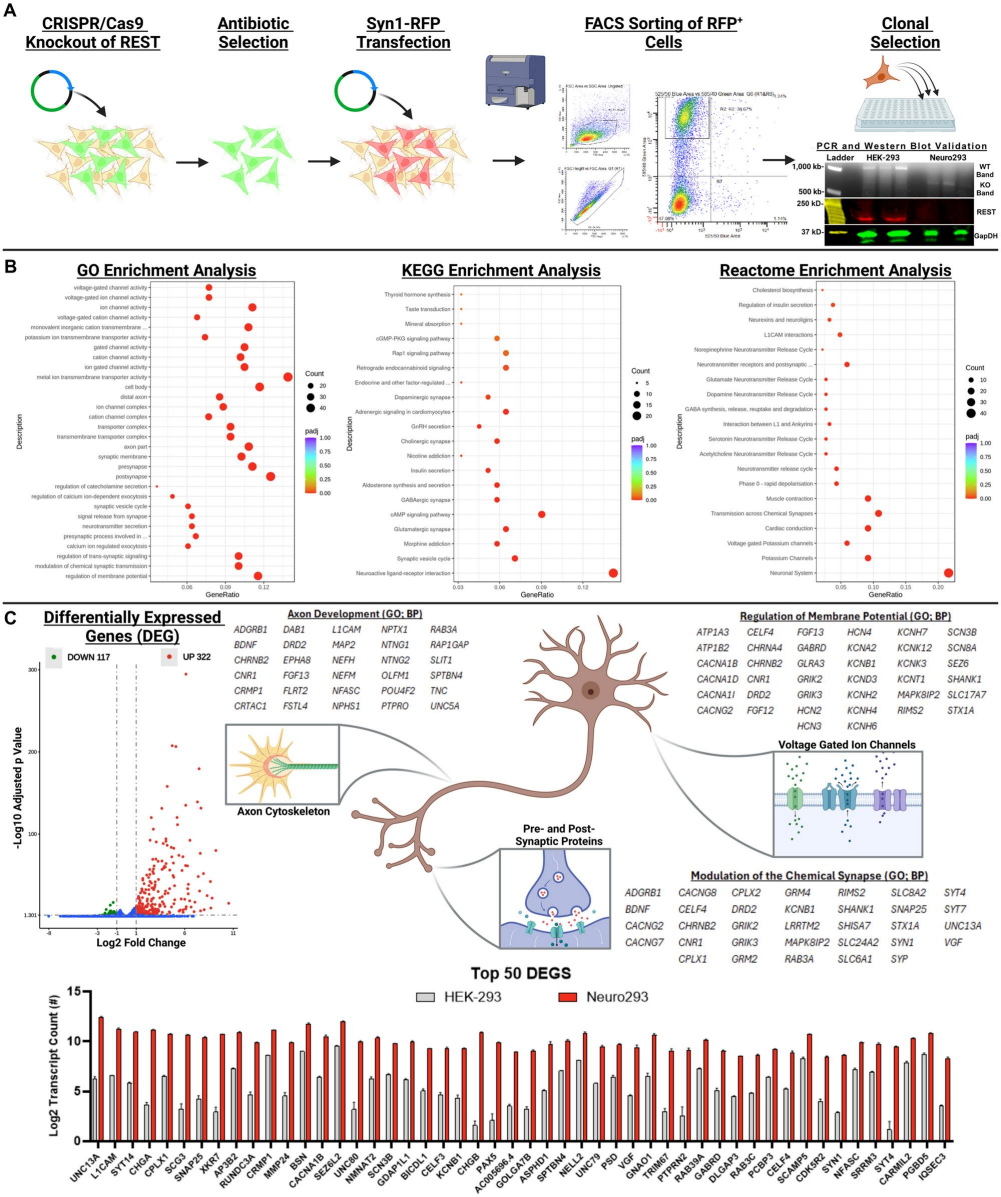
Establishing Neuro293’s using CRISPR/Cas9 to knockout REST upregulates neuronal-related gene transcription. CRISPR/Cas9 was used to knockout REST from HEK-239’s, and a single cell was isolated and expanded to establish Neuro293’s (A). PCR and western blot were performed on a single expanded clone (PCR = black and white bands; western blot = colored bands) (A). GO, KEGG, and Reactome enrichment analysis implicated a vast network of neuronal-related genes as upregulated following REST-KO (B) including genes associated with axon development, ion channels, and the regulation of the membrane potential, as well as pre-/post-synaptic functions, including neurotransmitter synthesis and release (C). All genes displayed in the Top 50 DEGS were significant discoveries after FDR correction. See Supplementary Materials 3 for high-resolution Dot Plots from (B). This figure was created with Biorender.com.

After re-establishing a population of REST-KO cells, single-cell clones were selected by diluting a sample of cells to 100 cells/ml and plating 10 µl of cell solution into single wells of a 96-well plate. All wells were evaluated for single cell attachments 24-h after seeding. Any well identified with more than a single cell was removed from further investigation. Once cells reached 70% confluence, all identified clones were screened for unintentional integration of either the CRISPR/Cas9 plasmid (green fluorescence) or mCherry plasmid (red fluorescence), and any colonies exhibiting integration were removed from analysis. Importantly, we detected a very low prevalence of cells propagating either eGFP or RFP before selection, suggesting a low rate of integrating plasmids into the host genome after transient transfection. In total, three singular clones were expanded and genotyped for successful knockout of the targeted region, and one was selected to expand (Fig. 1A).

Genotyping of cells used primers flanking the prospective knockout region (Forward: GAGTGCAGAGAAGCAGGCAA; Reverse: CACTCCACTTTGCTGTCACC). Genotyping was performed on cDNA derived from cellular RNA using Trizol. Successful knockout of the DNA binding domain of REST reveals a 790 bp band, while intact REST yields a 1128 bp band. A single clone with a validated single band at 790 bp was expanded and frozen in aliquots and will be referred to as Neuro293 (Fig. 1A).

Off-target CRISPR/Cas9 analysis was performed to determine the extent of unintentional mutations added to the genome. DNA was isolated from both HEK-293’s and Neuro293’s using DNeasy Blood and Tissue Kit (QIAGEN, Valencia, CA) and shipped to Novogene for sequencing and analysis. Both guide arms were utilized to determine potential off-target mutations. To determine the extent to which off-target mutations may have affected transcriptional response to REST-KO, we focused our analysis on identifying mutations to transcriptional exon regions as well as 5′- and 3′-untranslated regions (UTRs) of genes that were identified to be significantly up- or down-regulated by RNA-sequencing (see Methods section below) in Neuro293’s after false-discovery rate (FDR) correction. As recommended by Novogene, mutations matched to large areas of duplications as well as repeat regions are likely sequencing errors or false positives and were removed from analysis. Processed data on somatic mutations are provided in Supplemental materials (Supplemental Materials 2). Due to the excessively large file sizes, raw data have been saved on local hard drives and can be available upon request.

### RNA-Seq and bioinformatics

#### RNA isolation

RNA-sequencing, data quality control, and bioinformatics analyses were performed at Novogene. In total, three samples per group were used for RNA sequencing. cDNA library construction utilized poly-T oligo-attached magnetic beads to isolate messenger RNA, followed by the use of random hexamer primers for first-strand synthesis. Sequencing reads were processed for quality control before being processed for alignment using HISAT2 [11]. Raw read counts were normalized using the DESeq method, and differential gene analysis was performed using DESeq2, with FDR using the Benjamini–Hochberg method. Differentially regulated genes were identified based on having at least a 2-fold increase in expression (|log2(Fold Change)| ≥ 1) with an FDR-adjusted *P*-value of ≤ 0.05.

#### KEGG/GO/reactome pathway enrichment analysis

The list of differentially regulated genes was assessed using Kyoto Encyclopedia of Genes and Genomes (KEGG) pathways [12], Gene Ontology (GO) term enrichment, as well as Reactome pathway analyses [13]. Bioinformatics assessments were performed using the Bioconductor package clusterProfiler [14]. Processed RNA-sequencing data are available in supplemental materials (Supplemental Materials 3).

### Western blotting

Protein from cells was harvested (*n* = 3/group) using radioimmunoprecipitation assay (RIPA) lysis buffer (1×; 20–188; Millipore Sigma) containing protease inhibitors (cOmplete Mini; Millipore Sigma; 04693159001) and diluted to 1 µg/µl containing 4X Laemmli sample buffer (Bio-Rad; 1610747) and 2.5% beta-marceptoethanol. Samples were boiled for 5 minutes before loading onto 4%–15% pre-formed gradient acrylamide gels (Bio-Rad; 4561086) and separated with electrophoresis (70 mV for 30-min, then 120 mV until the front reached the bottom). Proteins were transferred onto nitrocellulose membranes using semi-dry transfer (Bio-Rad; 1704158). Membranes were dried to increase protein adherence before blocking in 5% non-fat milk in Tris-buffered saline (TBS) for 1-h at room temperature.

All antibody incubations were performed at room temperature overnight while rocking in TBS containing 0.1% Tween-20 (TBS/T). Primary antibodies included REST/NRSF (Sigma-Aldrich; 07-579; 1:2000), Syn1 (Cell Signaling Technologies; D5297S; 1:2000), Snap-25 (Sigma-Aldrich; S9684; 1:2000), Kv1.2 (DSHB Hybridoma; K14/16; K14/16 was deposited to the DSHB by Trimmer, J.S; used at 1:200), and Beta-Actin (Sigma-Aldrich; A2228) or GapDH (Sigma-Aldrich; G8795; 1:10 000) as loading controls. Antibody labeling was visualized using fluorescence detection and was targeted using goat anti-IgG IRDye 700/800 (LICORbio; 1:5000). For protein normalization, the highest Beta-Actin band amongst all samples was used to determine a loading ratio for each sample, and all other wells were normalized accordingly.

### Immunocytochemistry of neuron-specific proteins

Immunocytochemistry was performed to evaluate the distribution of neuronal proteins within Neuro293’s and compared to the relative expression against HEK-293’s. Cells were seeded on coverslips as described above and fixed using 2% formaldehyde. Cover slips were blocked for nonspecific protein binding using 5% normal goat serum for 1-h at room temperature, followed by incubation in primary antibodies. Antibody labeling pairs included Chicken anti-NFH (Aves Labs; NFH; 1:200) and Rabbit anti-Syn1 (Cell Signaling Technologies; D5297S; 1:200), as well as Chicken anti-β3-Tubulin (Aves Labs; TUJ; 1:200) and Rabbit anti-NeuN (Novus Biologics; NBP1-77686; 1:200). Primary antibodies were revealed using secondary antibodies, Goat anti-IgG Alexafluor 488/547 (Invitrogen; 1:500). Cells were imaged using confocal microscopy for cellular localization.

### Neuron-restricted promoter expression

To test the ability for Neuro293’s to activate neuron-restricted promoters commonly used for *in vivo* use, we performed transient plasmid transfections using Lipofectamine 3000 (1 µg DNA, 1 µl P3000, and 1 µL Lipofectamine per 12 well) into both HEK-293’s and Neuro293’s that were seeded on top of 12-mm round Poly-D-Lysine/Laminin coated cover slips. Plasmids used for transfection included Syn1-Cre-p2a-dTomato (Addgene; Addgene ID: 107738), CamK2a-RFP (Addgene; Addgene ID: 22908) [15], and HB9-eGFP (Addgene; Addgene ID: 16275) [16]. At 48 h post-transfection, cover slips were fixed in 2% formaldehyde solution prepared from paraformaldehyde (Sigma-Aldrich), stained with DAPI for nuclear identification, and mounted on slides with Epredia Immu-Mount (Fisher Scientific; 9990402). Images were obtained using conventional fluorescence microscopy, and all imaging parameters were maintained for all imaged regions and all samples. The total number of expressing cells per field of view was normalized to the total number of DAPI^+^ cells. Each cell’s fluorescence intensity was quantified.

Experiments to determine the ability of Neuro293’s to express under neuron-restricted promoters were then evaluated using a packaged AAV vector. In total, 1.0 × 10^9^ genome copies of Syn1-Cre-p2a-dTomato that were packaged into an AAV2-retro psuedotype was used to transduce either 1.0 × 10^5^ HEK-293’s or Neuro293’s seeded on top of coated coverslips in singular wells of 12 wells. Cells were evaluated at 3 days post-infection. Images were taken based on the observation of cells excited by a 565-nm LED light. Due to the observation of many small accumulations of autofluorescent pigments, we also captured images at 488-nm to discriminate dTomato signal from autofluorescence (autofluorescence is observed in both 565 and 488 nm, while dTomato is not observed at 488 nm). Observable cells in each image were counted for analysis and normalized to total DAPI counts in each image using QuPath. Of note, extremely low dTomato^+^ counts per total cell were observed, likely due to the extremely fast doubling time of HEK-293’s and Neuro293’s which dilutes out the total number of fluorescent cells due to AAVs being non-integrating.

### Excitability assessments

To assess membrane excitability, current was recorded in the whole-cell configuration of the patch-clamp technique. After achieving the whole cell configuration, a voltage protocol stepping from −80 to +40 mV in 5 mV increments was applied and current recorded. Upon whole-cell access, we waited approximately 2 min for equilibration of the pipette solution with the cell interior. Pipette and cell capacitance were compensated and up to 80% Rseries compensation was used. Current was recorded with the dPatch amplifier system (Sutter Instruments, Novato, California) and analyzed with IgorPro 8. Analog signal was filtered at 2 kHz and digitized at 10 kHz. Membrane current recordings were performed at room temperature (20–22°C). The pipette solution consisted of (in mmol/l) 130 mM KCl, 1 MgCl_2_, 5 EGTA, 5 Mg-ATP, 10 HEPES, pH 7.2. Recordings were conducted in Tyrode’s solution. Physiological Tyrode bath solution contained (in mmol/l) 140 NaCl, 5.4 KCl, 1.2 KH_2_PO_4_, 5 HEPES, 5.55 glucose, 1 MgCl_2_, and 1.8 CaCl_2_, pH 7.4. Recordings were made on n = 5 HEK-293’s and *n* = 5 Neuro293’s to measure ionic current. Peak outward currents were plotted as a function of the voltage step.

### Statistics

For statistical analysis of RNA-sequencing, see above. For all other data, Welch’s *t*-tests were performed to compare between-group differences. Western blot arbitrary values were square root transformed to fit assumptions prior to statistical analysis.

## Results

### RNA-sequencing demonstrates a differential expression of neuronal-related genes, including pre-/post-synaptic functions, voltage-gated ion channel regulation, and neurotransmission

Of 32,840 identified transcripts, 4,161 were significantly affected by REST-KO before FDR correction, with 1,561 persevering through FDR correction. Differentially expressed genes (DEG) were defined as significant post-FDR correction and possessing at least a 2-fold increase in gene expression, which identified a total of 439 mRNA transcripts. KEGG, GO, and Reactome analyses were performed on the 439 differentially expressed transcripts. In total, 322 DEG transcripts were upregulated and 117 were downregulated (Fig. 1C). Among the top identified, GO enrichment pathways implicated REST-KO in the regulation of membrane potential (BP; GO: 0042391), post- (CC; GO: 0098794) and pre-synaptic functions (CC; GO: 0098793), axon parts (CC; GO: 0033267), and metal ion transmembrane transporter activity (MF; GO: 0046873) (Fig. 1B). The most notable KEGG enrichment pathways included neuroactive ligand-receptor interactions (hsa04080), cAMP signaling pathway (hsa04024), synaptic vesicle cycle (hsa04721), and the glutamatergic synapse (hsa04724) (Fig. 1B). Finally, Reactome enrichment implicated the DEGs in neuronal systems (R-HSA-112316), potassium channels (R-HSA-1296071), transmission across the chemical synapse (R-HSA-112315), as well as cardiac conduction (R-HSA-5576891) (Fig. 1B). Taken together, knockout of REST from HEK-293’s modulates a wide network of neuronal-related genes to enable the expression of human neuron-restricted transcripts.

### Western blot reveals an upregulation of proteins known to be regulated by REST

**Figure 2.**
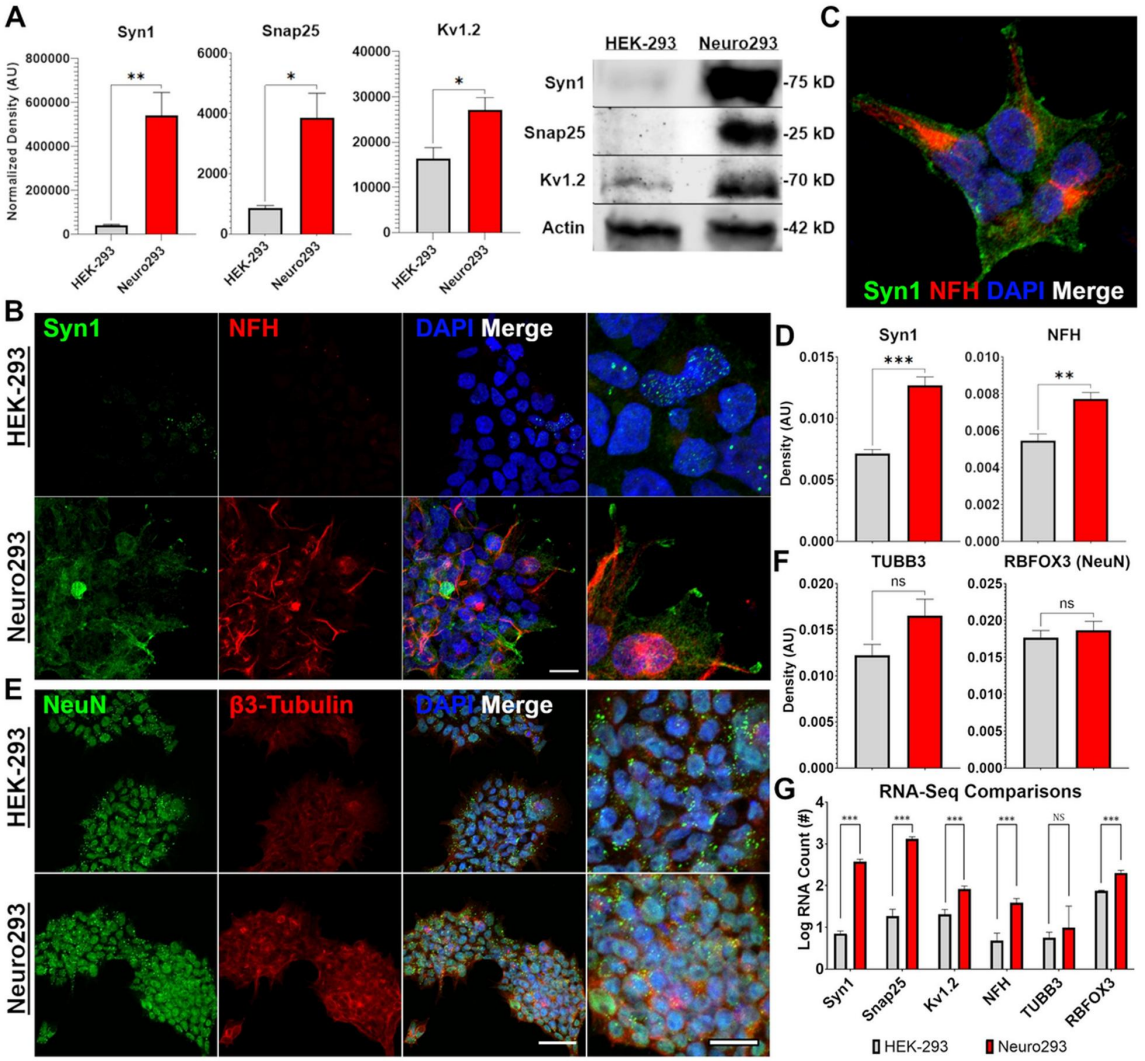
REST-KO stably upregulates neuron-restricted proteins, enabling the high-throughput biochemical interrogation of human neuronal proteins. Neuronal-specific genes Syn1 and Snap25, as well as the non-neuronal-specific ion channel Kv1.2, were upregulated at the protein level using western blot (A). Sub-cellular localization of Syn1 and NFH identifies what appears to be assembly and accumulation along a preferred cellular extension (B and C). Protein accumulation within Neuro293s and HEK-293s was closely predicted by RNA levels identified in RNA-sequencing (B, E, D–G). NeuN was already expressed in HEK-293s while TUBB3 displayed trends towards an increase that were not significant at either the protein or mRNA level. *n* = 3 replicates/group (A), *n* = 4–6 images/group (D and F). Scale Bars = 100 µm. Error Bars = SEM. *P <* *0.05, **P <* *0.01, ***P <* *0.001, ns = not significant.

Our western blot analysis demonstrated a significant upregulation of Syn1 (*t*_(4)_ = 4.72, *P *=* *0.009), Snap25 (*t*_(4)_ = 3.71, *P *=* *0.02), and Kv1.2 (gene name KCNA2) (*t*_(4)_ = 2.93, *P *=* *0.04), as predicted by RNA-sequencing. Surprisingly, despite Syn1 being considered a neuron-restricted gene, protein was identified in HEK-293’s, albeit at significantly lower levels, which is also consistent with RNA-sequencing results (HEK-293 *M* = 7.6; Neuro293 *M* = 376.6 transcript count). While Kv1.2 is not a neuron-restricted gene, we identified both an increased transcriptional expression (*P *<* *0.001; HEK-293 *M* = 22.3; Neuro293 *M* = 82.3 transcript count), as well as protein expression [17]. Collectively, we demonstrate that knockout of REST from HEK-293’s can be used to readily generate many mature neuronal proteins for the high-throughput interrogation of human neuronal protein biochemistry (Fig. 2A). Raw images of blots are available in Supplemental Materials 1.

### Immunocytochemistry of Syn1, NFH, β3-tubulin, and NeuN reveal sub-cellular preference of axonal markers to singular processes and synaptic proteins to the process terminals

Neuro293’s and HEK-293’s were labeled for either Syn1 and NFH, or NeuN and β3-Tubulin to evaluate the expression and localization of neuronal proteins. We observed a significant increase in fluorescence labeling for Syn1 (*t*_(8)_ = 6.17, *P *=* *0.0003) and NFH (*t*_(8)_ = 4.30, *P *=* *0.0026) within Neuro293’s that is consistent with RNA-sequencing (Fig. 2B-G). However, NeuN (RBFOX3) was not significantly upregulated (*t*_(8)_ = 0.65, *P *=* *0.52), nor was β3-Tubulin (TUBB3) which trended towards a modest increase in both ICC (*t*_(8)_ = 2.03, *P *=* *0.076) and mRNA (Fig. 2E–G). Syn1 expression accumulated at the ends of processes extending from Neuro293’s that was not observed in HEK-293’s (Fig. 2B). Instead, Syn1 expression was mostly absent in HEK-293’s except in seemingly random cells, where Syn1 was observed unorganized and speckled around the soma. NFH appeared to exert a preference for processes extending from Neuro293’s and often displayed a bias towards a single process (Fig. 2B and C). NFH staining was not observed in HEK-293’s (Fig. 2B). Our findings point towards a neuron-like polarization and sub-cellular compartmentalization of neuronal-specific proteins in Neuro293’s and imply the use of REST-KO to interrogate mechanisms of cytoskeletal assembly and synaptic connections.

Importantly, while the polarized and sub-cellular localization of axonal and synaptic proteins did display consistencies with early neuronal development, we do not observe a robust difference in gross morphology in normally dividing cells after REST-KO. Neuro293’s still grow in clusters and send small processes extending from their soma (Fig. 2). Further, we performed a doubling-time assessment of both HEK-293’s (22.37 ± 2.10 h; M ± SD; *n* = 3) and Neuro293’s (20.77 ± 1.62 h; M ± SD; *n* = 4) and did not detect differences in proliferation rates (*t*_(5)_= 0.79; *P *=* *0.46).

### Reporter gene expression through the Syn1 promoter is upregulated after AAV transduction in Neuro293’s, but paradoxically downregulated after transient plasmid transfection

**Figure 3.**
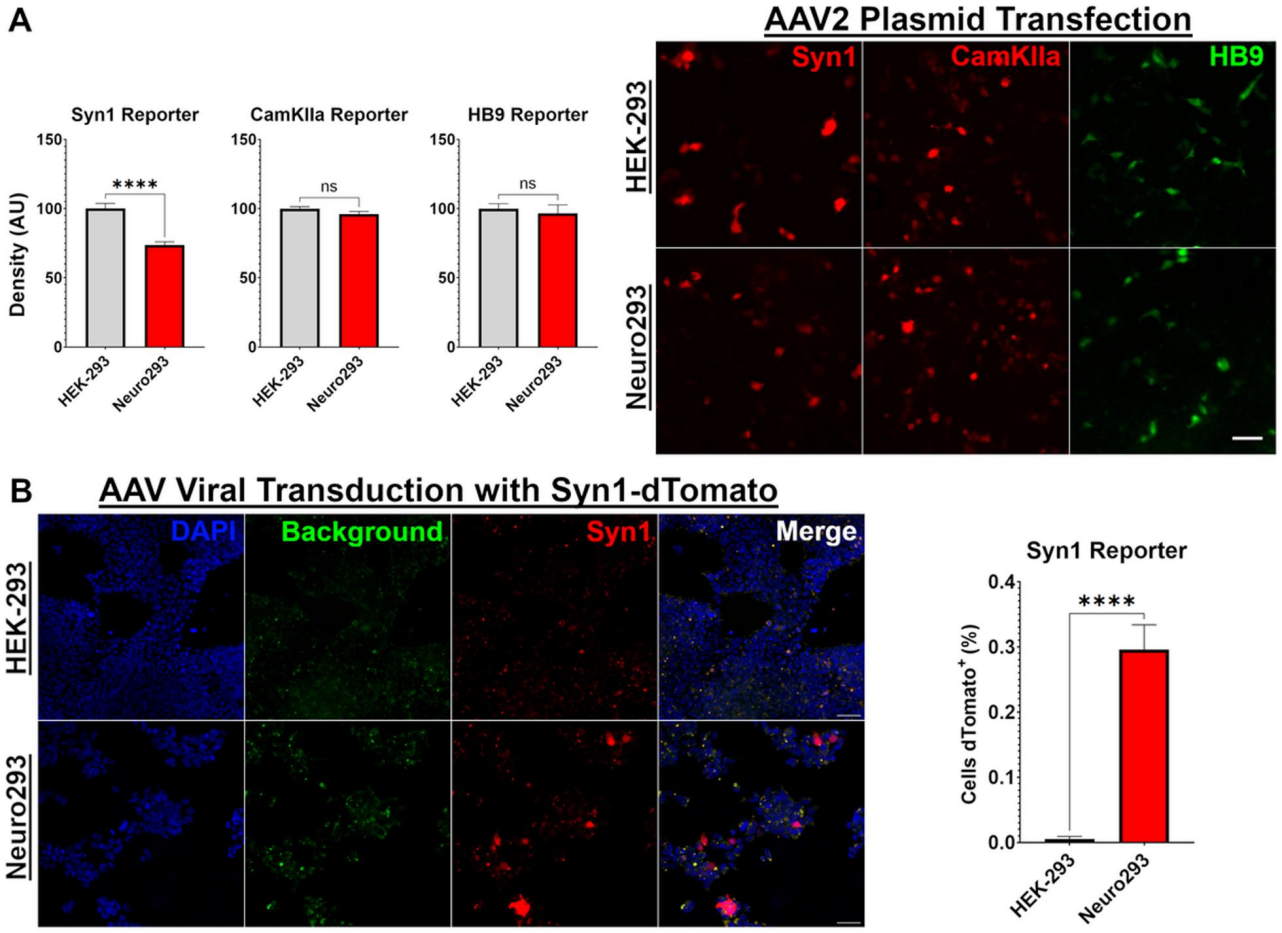
Transient transfection with plasmids enables protein expression in a promotor-independent manner through AAV2 plasmids, but only Neuro293’s express through the Syn1 promoter after viral transduction. Neuron-restricted promoters Syn1, CamKIIa, and HB9 were tested for the ability to express downstream proteins after transient transfection with AAV2 plasmids **(A)**. Surprisingly, all promoters exhibited reporter-gene expression in both Neuro293 and HEK-293 cells, even when RNA-sequencing indicated an absence of RNA-transcriptions (CamKIIa was not expressed in either cell, and Syn1 was lowly expressed in HEK-293’s; Supplementary Table 3). Paradoxically, reporter gene expression was less active in Neuro293’s for currently unknown reasons, despite a higher level of Syn1 mRNA and protein expression (Figs 1 and 2). Use of packaged AAV vectors to transduce cells, in contrast, demonstrates an expected higher expression of reporter genes through the Syn1 promoter (B). *n* = 6–8 images/group. Scale Bars = 100 µm. Error Bars = SEM. ****P <* *0.0001, ns = not significant.

The capacity to express proteins through neuron-restricted promoters that are commonly used for *in vivo* gene transfer was a large reason to develop the Neuro293 cell line. Much to our surprise, we observed a robust reporter gene expression after transient transfection with plasmids that used neuron-restricted promoters, including the Syn1, CamK2a, and HB9 promoters. Further, we observed a decrease in reporter gene expression from the Syn1 promoter in HEK-293’s compared to Neuro293’s (*t*_(12)_ = 5.95, *P < *0.0001), but did not observe differences through CamK2a (*t*_(13)_ = 1.69, *P *=* *0.11) or HB9 (*t*_(12)_ = 5.18, *P *=* *0.613). Unlike the mRNA expression of Syn1 (HEK-293 M = 7.18; Neuro293 M = 381.9 transcript count), neither HEK-293’s nor Neuro293’s express CamK2a mRNA (HEK-293 *M* = 0.0; Neuro293 *M* = 1.27 transcript count), while both cells express comparable transcriptional levels of MNX1 (HB9) (HEK-293 *M* = 827.07; Neuro293 *M* = 887.37 transcript count). The comparable expression, or higher expression, of reporter genes through all promoters, even in HEK-293’s suggests alternative transcriptional mechanisms mediate leaky expression post-transfection of plasmids on AAV backbones, potentially through ITR regions [18, 19] (Fig. 3A).

While it is interesting and potentially very useful to know that plasmid transfection can lead to transcriptional activity independent of promoter selection, it remained important to evaluate promoter activity specifically. To determine if we would elicit a differential response between plasmid transfection and viral transduction, we next utilized AAV particles directly to compare transgene expression. As expected, there was a significant increase in reporter gene expression through the Syn1 promoter in Neuro293’s when cells were transduced directly with viral particles (*t*_(14)_ = 7.52, *P *<* *0.0001; Fig. 3B).

Collectively, our results validate the ability for Neuro293’s to express proteins through neuron-restricted promoters after viral transduction, but also suggest that alternative mechanisms within AAV plasmids may facilitate unspecific transcription through mechanisms independent of intended promoter selection. Such findings have important implications for interpreting leaky expression of transiently transfected plasmids *in vitro*.

### Membrane excitability does not increase under patch-clamp despite an increase in mRNA and protein expression

**Figure 4.**
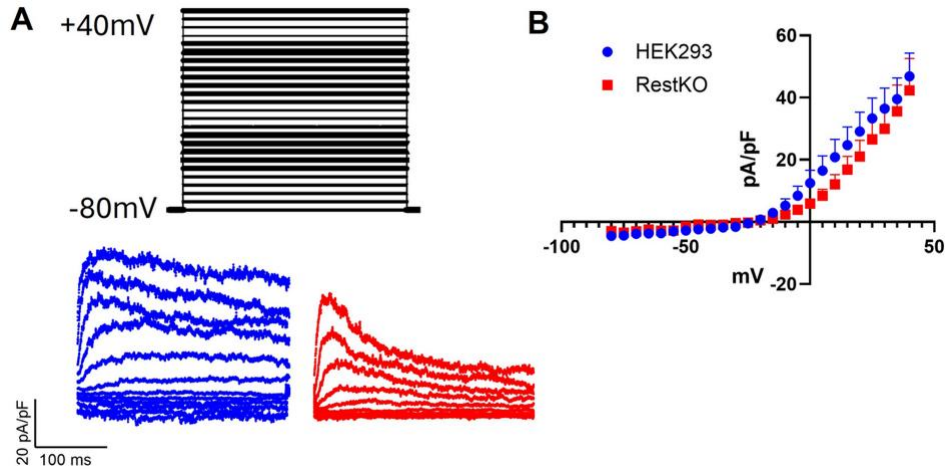
REST-KO does not augment membrane excitability despite an upregulation of voltage-gated ion channels. Patch clamp on HEK-293’s and Neuro293’s was used to test membrane excitability through an ascending range of voltage (A and B). Representative family of current traces, Vtest ranging from −80 to +40 in 10-mV increments, with voltage protocol schematic shown above (A). Current/voltage curve fails to show differences in current density between REST-KO and HEK-293’s (B). *n* = 5 HEK-293 and *n* = 5 Neuro293 cells/group. Error Bars = SEM.

While we have demonstrated a significant utility of Neuro293’s for use to study neurobiology and neural biochemistry *in vitro*, the ability to further evaluate membrane excitability remains to be determined. Despite observing a significant upregulation of voltage-gated ion channels at both the level of mRNA and protein (for Kv1.2), we did not observe a difference in membrane excitability in voltage patch-clamp experiments (Fig. 4). It is therefore not recommended to use Neuro293’s to study membrane excitability without further modifications.

### Whole-genome sequencing description of potential off-target mutations

Due to known off-target effects caused by CRISPR–Cas9, we performed whole-genome sequencing on HEK-293’s and Neuro293’s (Novogene Co.) to evaluate the extent to which transcripts upregulated in our RNA-sequencing dataset may have been affected by CRISPR–Cas9-mediated mutations. We specifically chose to report somatic mutations occurring within the exon regions as well as 5′- and 3′-UTRs.

Across the entire genome, large structural variants defined as greater than 50 bp mutations included 1 insertion and 182 deletions observed between Neuro293’s and HEK-293’s. Amongst numerous smaller insertions/deletions (<50 bp; 7747 mutations in total, most occurring in intergenic and non-coding regions), a total of 34 were observed within protein-coding reading frames and 112 occurred within the 5′- and 3′-UTRs. Total short nucleotide polymorphism (SNPs) included 20,677 identified differences, of which 290 were observed in protein-coding regions and 281 within the 5′- and 3′-UTRs (See Table 1 for a more detailed overview, or Supplemental Materials 2).

**Table 1.** Off-Target Analysis of Crispr–Cas9-mediated REST-knockout.

Total identified mutations		Upregulated genes carrying exonic mutations in Neuro293					Upregulated genes carrying UTR mutations in Neuro293				
Mutation type	Identified mutations (#)	Gene name	Mutation Location	Reading frame effect	Mutation type	Expression	Gene name	Mutation location	Reading frame effect	Mutation type	Expression
**Structural variants** *(>50bp)*		POU4F2	Exonic	Synonymous	SNP	UP	GALNT13	UTR5	Breakpoint	SV	UP
**Total**	**2755**	NPTXR	Exonic	Synonymous	SNP	UP	NSD1	UTR3	BND	SV	DOWN
**INS**	**1**	NEFH	Exonic	Synonymous	SNP	UP	ONECUT2	UTR5	BND	SV	UP
**DEL**	**182**	CHST1	Exonic	Synonymous	SNP	UP	MADD	UTR5	BND	SV	UP
INV	185	CRNDE	Exonic	Synonymous	SNP	UP	ATP1B2	UTR5	BND	SV	UP
DUP	57	CCNE1	Exonic	Synonymous	SNP	DOWN	WASF3	UTR5	BND	SV	UP
BND	2330	TRIM25	Exonic	Synonymous	SNP	DOWN	LYSMD4	UTR5	BND	SV	DOWN
**Small insertions/deletions** *(<50bp)*		POLR1A	Exonic	Synonymous	SNP	DOWN	BMP2	UTR3	.	SNP	DOWN
**Total**	**7747**	KCNS2	Exonic	Synonymous	SNP	UP	CHRM4	UTR3	.	SNP	UP
**CDS**	**34**	NFASC	Exonic	Missense	SNP	UP	DISP3	UTR3	.	SNP	UP
frameshift deletion	17	CHD5	Exonic	Missense	SNP	UP	HMBOX1	UTR3	.	SNP	UP
frameshift insertion	9	KCNA2	Exonic	Missense	SNP	UP	PAX2	UTR3	.	SNP	UP
non-frameshift deletion	7	LARGE2	Exonic	Missense	SNP	UP	PI15	UTR3	.	SNP	UP
non-frameshift insertion	0	CNTFR	Exonic	Missense	SNP	UP	SPIRE2	UTR3	.	SNP	UP
stopgain	1	MVP	Exonic	Missense	SNP	UP	SULT1C4	UTR3	.	SNP	DOWN
stoploss	0	CHAF1B	Exonic	Missense	SNP	DOWN	TEX22	UTR3	.	SNP	DOWN
unknown function	0	RBM15B	Exonic	Missense	SNP	DOWN	UFD1	UTR3	.	SNP	UP
**5′-UTR**	**3**	C1GALT1C1	Exonic	Missense	SNP	UP	WDFY1	UTR3	.	SNP	UP
**3′-UTR**	**109**	ZNF608	Exonic	Missense	SNP	DOWN	CRMP1	UTR5	.	SNP	UP
**Other**	**7596**	SYNJ1	Exonic	Missense	SNP	UP	GNAO1	UTR5	.	SNP	UP
Intronic	3122	TTK	Exonic	Missense	SNP	UP	HSF4	UTR5	.	SNP	UP
Splicing	3	CELSR3	Exonic	Missense	SNP	UP	MMP15	UTR5	.	SNP	UP
ncRNA_exonic	18	ATP8B3	Exonic	Missense	SNP	UP	KCNB1	UTR3	.	Insertion/Deletion	UP
ncRNA_intronic	491	NCOA2	Exonic	Missense	SNP	DOWN	CLVS2	UTR3	.	Insertion/Deletion	UP
ncRNA_splicing	1	PAK4	Exonic	Missense	SNP	UP	FAM155A	UTR3	.	Insertion/Deletion	UP
Upstream	41	NCKAP5L	Exonic	Missense	SNP	DOWN	ZFHX3	UTR3	.	Insertion/Deletion	DOWN
Downstream	58	ACSS1	Exonic	BND	Structural Variant	UP	STAG1	UTR3	.	Insertion/Deletion	DOWN
Intergenic	3862	MGAT5B	Exonic	BND	Structural Variant	UP	CDK5R1	UTR3	.	Insertion/Deletion	UP
**SNPs** *(1 bp)*		NAT16	Exonic	BND	Structural Variant	UP	TMEM65	UTR3	.	Insertion/Deletion	UP
**Total**	**20677**	OLFM2	Exonic	BND	Structural Variant	UP	NKX2-5	UTR3	.	Insertion/Deletion	DOWN
**CDS**	**290**	RREB1	Exonic	BND	Structural Variant	DOWN	ZBTB6	UTR3	.	Insertion/Deletion	UP
Synonymous	89	SOGA3	Exonic	BND	Structural Variant	UP	ZXDB	UTR3	.	Insertion/Deletion	DOWN
Missense	185	SSR4	Exonic	BND	Structural Variant	UP	AP3S1	UTR3	.	Insertion/Deletion	UP
Stopgain	14	IPO7	Exonic	BND	Structural Variant	UP					
Stoploss	0	CACNA1I	Exonic	BND	Structural Variant	UP					
Unknown function	2	FBXO41	Exonic	Breakpoint	Structural Variant	UP					
**5′-UTR**	**70**	SPR	Exonic	Breakpoint	Structural Variant	UP					
**3′-UTR**	**211**										
**Other**	**20096**					**Table Key**					
Intronic	7318			INS: The number of Insertions.			CDS: Insertions/deletions in coding region.				
Splicing	7			DEL: The number of deletions.			ncRNA: Non-coding RNAs.				
ncRNA_exonic	84			INV: The number of inversions.			Splicing: Insertion/deletion/SNP within 2-bp of a splicing junction.				
ncRNA_intronic	1290			DUP: The number of tandem duplications.			Upstream: Insertion/deletion/SNP within 1 kb away from transcription start site.				
ncRNA_splicing	3			BND: The number of translocations.			Downstream: Insertion/deletion/SNP within 1 kb away from transcription end site.				
Upstream	163			UTR: Untranslated Region.			SNP: Short nucleotide polymorphism.				
Downstream	147										
Intergenic	11084										

Note: Whole-genome sequencing was performed on our HEK-293 and Neuro293 cell lines and mutation differences were compared between both genomes. Structural variants consisting of greater than 50 bp represent large insertions, deletions, or shifting of gene sequences. Small insertions/deletions consisting of less than 50 bp include mutations affecting the protein coding sequences (CDS), the 5′- and 3′-UTR of mRNA, as well as mutations occurring in other regions such as intergenic locations or intronic regions. Similar analyses were performed on individual SNPs that reflect a single bp difference. Mutations from structural variants, small insertions/deletions, and SNPs are presented from whole-genome analysis. Data from RNA-sequencing that identified genetic transcripts which were significantly affected by REST-KO were aligned to mutations identified in exonic or 5′/3′-UTR regions. In total, 70 of the 1561 genes affected by REST-KO contained mutations within the exonic or 5′/3′-UTR regions.

Gene transcripts that were identified as significantly up- or down-regulated after REST-KO were aligned with genes containing identified mutations to help predict the possibility that genetic perturbations may account for expression differences observed in our RNA-seq data. In total, 70 out of 1,561 (∼4.5%) genes significantly affected by REST-KO displayed mutations in Neuro293 cells when using the HEK-293 genome as a baseline. Among all 70 mutations, 37 affected exon coding sequences uniquely, 26 affected only the 5′/3′-UTR region, and 7 larger structural variants affected both the exon and UTR regions. Of mutations affecting the exon, 9 SNPs led to synonymous changes within the protein coding region, but 17 SNPs induced missense mutations, and 11 larger structural variants were observed as either breakpoint mutations or as translocations (Table 1). Importantly, the overwhelming majority of genes (>95%) significantly affected by REST-KO did not contain mutations within the assessed regions. While REST mRNA was not detected as significantly affected after REST-KO, likely due to the overall low abundance, the genome of REST was confirmed to contain deletion and breakpoint mutations occurring within exonic regions, as anticipated.

## Discussion

Our results support the ability for REST-KO in HEK-293’s to upregulate mature neuron-restricted proteins for the interrogation of neural biochemistry in a high-throughput manner. Further, we demonstrate that neuron-restricted promoter activity can be achieved after viral transduction with AAVs, enabling the ability to assess promoter activity *in vitro*. We demonstrate that REST-KO upregulates a complex transcriptional profile of neuronal-related genes, even those which are not known to be neuronal specific, such as Kv1.2 [17].

Obtaining mature neuronal proteins, particularly of human origin, remains a challenging and time-consuming process. By knocking out REST from HEK-293’s we demonstrate the ability to produce and obtain cells that retain mitotic ability but actively produce mature neuronal proteins. We chose to assess several proteins that are known to be expressed mainly in mature neurons to determine their expression in both western blot and immunocytochemistry. For western blot, we evaluated Syn1 and Snap25, which are pre-synaptic proteins known to be regulated directly by REST to validate the phenotype [20, 21]. We also evaluated the potassium channel Kv1.2, which is not a neural-specific protein, but displayed an upregulation in our RNA-sequencing results. The rationale for evaluating Kv1.2 was mostly associated with other ongoing experiments in our lab and the subsequent availability of antibodies, but also provided an ability to assess how well mRNA predicts protein expression.

For immunocytochemistry, we chose to evaluate Neurofilament 200 kDa and Syn1 due to their known expression only in mature neurons, as well as to determine the localization within the cells. Neurofilament 200 kDa is primarily localized to the axon in mature neurons. Similarly, Syn1 is found in the pre-synaptic terminal. Observing both Neurofilament 200 kDa and Syn1 accumulating in processes extending from Neuro293’s was a surprise finding that may provide a unique ability to study cell polarization as well as the assembly of the axon cytoskeleton, despite not observing gross morphological differences. Similarly, while β3-Tubulin and NeuN were not significantly upregulated, both proteins are predominatly neuronal in nature. β3-Tubulin is part of the neuronal cytoskeleton, while NeuN (RBFOX3) is a transcription factor predominantly expressed in neurons. Observing natural expression of NeuN and β3-Tubulin in HEK-293’s without REST-KO was unanticipated and eludes to prior findings that reported some neuronal-related proteins are expressed in HEK-293’s [22].

While our approach is certainly not the first method established to increase the efficiency of studying neurobiology *in vitro*, current protocols utilize immature, neuron-like cells such as the SH-SY5Y [3–5, 23]. Immortalized neuron-like cells have the advantage of being able to differentiate into neurons with typical neuronal morphology and electrophysiological properties. As a disadvantage, the differentiation of immortalized neuron-like cells into mature neurons still takes weeks of culture, are very sensitive to culture conditions, require expensive culture reagents, require standardization and demonstration of efficient differentiation, and can be difficult to transfect relative to HEK-293’s. While Neuro293’s are not neurons and do not exhibit excitable properties, the perpetual expression of mature neuronal proteins, ease of transfection and expansion, and relatively cheap culture reagents needed to propagate the cell line are advantages for the study of neurobiology. Conceptually, the use of REST-KO to elicit the forced upregulation of mature neuronal proteins may be able to be applied to other neuron-like cells to expedite *in vitro* experiments, which was not explored in our current work.

While it was not necessarily surprising to observe an increase in neuron-restricted proteins after REST-KO, observing what appeared to be a polarization of Neuro293’s to accumulate axonal proteins, such as NFH and Syn1, within the cellular protrusions was not anticipated. During development, neurons establish a polarized phenotype that directs axonal and pre-synaptic proteins towards a single projection [24]. While concrete evidence of cellular polarization of Neuro293’s was not established in this work, we do observe the assembly of neurofilaments such as NFH with what appears to be the accumulation of Syn1 at the tips of cellular protrusions. The utility for such observations has not yet been established.

Our biggest reason for establishing the Neuro293 cell line was to be able to test neuron-restricted promoters and downstream open reading frames (ORF) *in vitro* prior to packaging into viral particles for use *in vivo*. Ironically, our work demonstrates that the ORF can be transcribed through alternative mechanisms other than the inserted promoter sequences when AAV2 plasmids are used for transient transfection. On one hand, this suggests that neurons or Neuro293’s are not needed to test the ORF on AAV2 backbones; on the other, this can complicate a deeper understanding of promoter efficacy. For example, we have observed in ongoing work (unpublished data) that transduction of an AAV2 plasmid into Neuro293’s that utilize the TRE promoter from doxycycline-inducible expression systems, leads to ORF expression without co-expression of the TETON transactivator proteins. These observations suggest that alternative mechanisms on AAV2 plasmids initiate transcription, likely the ITR regions [18, 19, 25].

When we evaluated Syn1 promoter-driven transgene expression using packaged AAV vectors, we did not observe a robust expression of reporter genes in HEK-293’s but did observe expression in Neuro293’s. The mechanisms behind observing differences between plasmid and viral delivery of DNA on transgene expression are not understood but may be partially explained by differences in ITR confirmation between plasmid and viral DNA [25]. Our collective observations do support the ability for Neuro293’s to express neuron-restricted promoters but have also determined that unmodified HEK-293’s will be sufficient to test ORF expression using transient-transfection methods with AAV2 plasmids. To test the function of a neuron-restricted promoter, it therefore may be necessary to utilize either packaged viral vectors or utilize transient transfection of expression plasmids lacking ITR regions, rather than AAV2 plasmids.

## Study limitations

There are limitations and unaddressed questions from the work presented above. First, while we revealed a massive transcriptional shift in RNA-sequencing, it remains impossible to evaluate the protein production from each individual transcript. While several of the proteins we did assess are known to be regulated by REST, others are not directly associated, suggesting that REST-KO elicits a more complex direct, or indirect, effect across a breadth of the human genome, which was not deeply analyzed in this manuscript. Next, the extent to which CRISPR/Cas9 elicited off-target effects that changed the transcription of some of these proteins remains possible. Exactly which transcripts would be affected remain unknown, despite performing whole-genome analyses.

While we did use Syn1 expression of mCherry to select for cells using FACs sorting, we later demonstrated that the ITRs from the AAV2 plasmids are sufficient to drive Syn1 expression regardless of REST-KO, making this entire selection process unnecessary to obtain a REST-KO cell. Future experiments could focus on using either viral-mediated transduction or transfection with regular expression plasmids that do not contain ITR regions.

While the utility of Neuro293’s to study mature neuronal proteins is evident, a major limitation to this cell line, as of now, is a lack of membrane excitability. HEK-293’s are known to possess limited-to-absent excitable membranes, and do not elicit action potentials without forcing the expression of ion channels such as Kir2.1 [26]. Despite observing a significant upregulation of several voltage-gated ion channel transcripts and proteins in Neuro293’s, we did not detect a significant increase in voltage-gated K^+^-current. We speculate that Neuro293 cells fail to properly process or traffic ion channel proteins. Thus, further modifications may be needed to generate an excitable membrane, and if successfully obtained, the usefulness of Neuro293’s would expand significantly. While not being able to use Neuro293’s directly as an excitable cellular source, these observations may pose additional utility to use Neuro293’s to study ion channel regulation, trafficking, and/or membrane insertion.

Finally, while our whole-genome sequencing and off-target analyses demonstrated a significant number of mutations in Neuro293’s compared to HEK-293’s, it remains important to consider that many of the observed differences could stem from the selection process itself. Mutations are known to accumulate in dividing cells *in vitro*, leading to heterogeneity in the proliferating cell population. Our selection process isolated a single cell with REST-KO for expansion and compared the genome against the likely heterogeneous HEK-293 population. In hindsight, to deeply assess the extent that REST-KO exerted off-target effects, a better design would have been to select a single cell prior to REST-KO to reduce heterogeneity within the HEK-293 population. Regardless, data from whole-genome sequencing of HEK-293’s and Neuro293’s identified genes that may have possibly been transcriptionally affected by off-target CRISPR–Cas9 effects as opposed to REST-KO itself. We identified only a few genes that may have been affected by Crispr-Cas9 from our differential expression analysis, including up to 70 out of 1,561 total gene candidates (∼4.5%), of which only a few were relevant as partial contributors to our main conclusions (KCNA2, KCNS2, KCNB1, CACNA1L, NFASC). Of the mutations occurring in these genes, most were identified as small missense or synonymous SNPs, which likely exert less of an effect on transcriptional regulation as much as downstream protein function, if any at all. Insertion/deletion mutations occurring in the 5′/3′-UTRs may affect transcriptional regulation in unpredictable ways. While observing an abundance of total mutations is not out of line with prior reports of off-target effects of the CRISPR–Cas9 system [27], the identified abundance and alignment of affected genes do not change our major conclusions.

## Conclusions

In conclusion, we established a REST-KO HEK-293 cell line, named Neuro293’s, which enables the constitutive expression of mature neuronal proteins in a quickly expandable and easily transfectable cell line. Use of Neuro293’s may help expedite the interrogation of human neuronal biochemistry through several areas of neuronal functions, including the axonal cytoskeleton, pre- and post-synaptic protein production, systems of neurotransmitter synthesis and release, as well as the production of voltage-gated ion channels. However, despite upregulating voltage-gated ion channels, Neuro293’s still do not possess an excitable membrane. Collectively, our work establishes a novel tool to help expedite and simplify the production of mature neuronal proteins *in vitro*.

Highlights

- Knockout of REST in HEK-293’s upregulates mature neuronal proteins.
- REST-KO in HEK-293’s enables transgene expression through the Syn1 promoter.
- Despite an upregulation of voltage-gated ion channels, REST-KO in HEK-293’s does not increase membrane excitability.

## Supplementary Material

bpaf036_Supplementary_Data

## Data Availability

Processed RNA-sequencing data and bioinformatics analyses are available as supplementary materials. Any/all other data can be made available upon request.
