# Supplementary material for: Neuro293: A REST-knockout HEK-293 cell line enables the expression of neuron-restricted genes for the high-throughput testing of human neurobiology and the biochemistry of neuronal proteins
Source: Biol Methods Protoc. 2025 May 10;10(1):bpaf036. doi: 10.1093/biomethods/bpaf036 (PMC12202048; doi:10.1093/biomethods/bpaf036)
Supplement: bpaf036_Supplementary_Data [file bpaf036_supplementary_data.zip › Supplemental Materials 3 RNA Sequencing.zip!/Supplemental Materials 3 RNA Sequencing/Supplementary Materials 3 Bioinformatics Plots.zip!/RESTKOvsWT.all_DisGeNETdot.pdf]

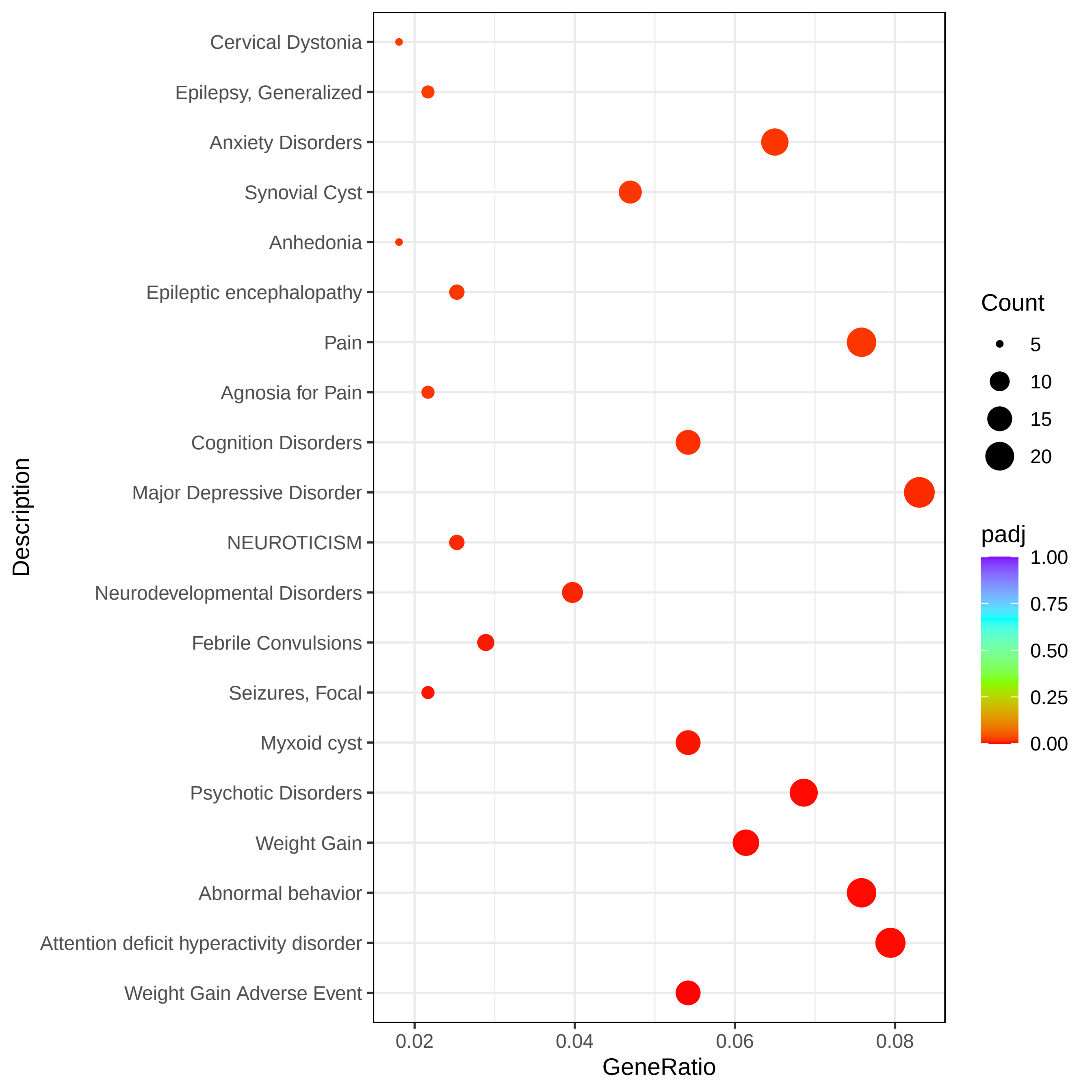

Cervical Dystonia
Epilepsy, Generalized
Anxiety Disorders
Synovial Cyst
Anhedonia
Epileptic encephalopathy
Pain
Agnosia for Pain
Cognition Disorders
Major Depressive Disorder
NEUROTICISM
Neurodevelopmental Disorders
Febrile Convulsions
Seizures, Focal
Myxoid cyst
Psychotic Disorders
Weight Gain
Abnormal behavior
Attention deficit hyperactivity disorder
Weight Gain Adverse Event
Description
0.02
0.04
0.06
0.08
GeneRatio
Count
5
10
15
20
padj
1.00
0.75
0.50
0.25
0.00
