# Supplementary material for: Neuro293: A REST-knockout HEK-293 cell line enables the expression of neuron-restricted genes for the high-throughput testing of human neurobiology and the biochemistry of neuronal proteins
Source: Biol Methods Protoc. 2025 May 10;10(1):bpaf036. doi: 10.1093/biomethods/bpaf036 (PMC12202048; doi:10.1093/biomethods/bpaf036)
Supplement: bpaf036_Supplementary_Data [file bpaf036_supplementary_data.zip › Supplemental Materials 1 REST KO sequence and Western Blots.zip!/Supplemental Materials 1 REST KO sequence and Western Blots/Supplemental Materials 1 Rest-KO Sequence.pdf]

Homo sapiens RE1 silencing transcription factor (REST), RefSeqGene on chromosome 4

gi|340139043|ref|NG_029447.1|

Sequence View

```
      1234567890123456789012345678901234567890123456789012345678901234567890
 7470 TGAGGAGGCACCACATGCCAGTGGCTGAATGATGAATGAGTTGTGCAAACAATTGGTAGAATTGTTTTAT
 7540 AGGGATAGTAGAAATTTTTTTTTGGTGATTGCAGTAGACTATTGGGAATTAACAACTATTTTAAAGATTT
 7610 GTTTTTAATAGGCGATGAAGTTTACTGAGTTATAAAGATCATACTGGAAAATTTTTAAGTTATGAAGAAT
 7680 TACAGCGATGTGGTTTTAAGCCAGTAACAGTAGTGTTTAAACTGGTGCTCTTGTTTTGTTTTGTTTTTAA
 7750 TTCAGAATACAGTTATGGCCACCCAGGTAATGGGGCAGTCTTCTGGAGGAGGAGGGCTGTTTACCAGCAG
                     M  A  T  Q  V  M  G  Q  S  S  G  G  G  G  L  F  T  S  S
 7820 TGGCAACATTGGAATGGCCCTGCCTAACGACATGTATGACTTGCATGACCTTTCCAAAGCTGAACTGGCC
        G  N  I  G  M  A  L  P  N  D  M  Y  D  L  H  D  L  S  K  A  E  L  A
 7890 GCACCTCAGCTTATTATGCTGGCAAATGTGGCCTTAACTGGGGAAGTAAATGGCAGCTGCTGTGATTACC
        A  P  Q  L  I  M  L  A  N  V  A  L  T  G  E  V  N  G  S  C  C  D  Y
 7960 TGGTCGGTGAAGAAAGACAGATGGCAGAACTGATGCCGGTTGGGGATAACAACTTTTCAGATAGTGAAGA
      L  V  G  E  E  R  Q  M  A  E  L  M  P  V  G  D  N  N  F  S  D  S  E  E
 8030 AGGAGAAGGACTTGAAGAGTCTGCTGATATAAAAGGTGAACCTCATGGACTGGAAAACATGGAACTGAGA
        G  E  G  L  E  E  S  A  D  I  K  G  E  P  H  G  L  E  N  M  E  L  R
 8100 AGTTTGGAACTCAGCGTCGTAGAACCTCAGCCTGTATTTGAGGCATCAGGTGCTCCAGATATTTACAGTT
        S  L  E  L  S  V  V  E  P  Q  P  V  F  E  A  S  G  A  P  D  I  Y  S
 8170 CAAATAAAGATCTTCCCCCTGAAACACCTGGAGCGGAGGACAAAGGCAAGAGCTCGAAGACCAAACCCTT
      S  N  K  D  L  P  P  E  T  P  G  A  E  D  K  G  K  S  S  K  T  K  P  F
 8240 TCGCTGTAAGCCATGCCAATATGAAGCAGAATCTGAAGAACAGTTTGTGCATCACATCAGAGTTCACAGT
        R  C  K  P  C  Q  Y  E  A  E  S  E  E  Q  F  V  H  H  I  R  V  H  S
 8310 GCTAAGAAATTTTTTGTGGAAGAGAGTGCAGAGAAGCAGGCAAAAGCCAGGGAATCTGGCTCTTCCACTG
        A  K  K  F  F  V  E  E  S  A  E  K  Q  A  K  A  R  E  S  G  S  S  T
 8380 CAGAAGAGGGAGATTTCTCCAAGGGCCCCATTCGCTGTGACCGCTGCGGCTACAATACTAATCGATATGA
      A  E  E  G  D  F  S  K  G  P  I  R  C  D  R  C  G  Y  N  T  N  R  Y  D
 8450 TCACTATACAGCACACCTGAAACACCACACCAGAGCTGGGGATAATGAGCGAGTCTACAAGTGTATCATT
        H  Y  T  A  H  L  K  H  H  T  R  A  G  D  N  E  R  V  Y  K  C  I  I
 8520 TGCACATACACAACAGTGAGCGAGTATCACTGGAGGAAACATTTAAGAAACCATTTTCCAAGGAAAGTAT
        C  T  Y  T  T  V  S  E  Y  H  W  R  K  H  L  R  N  H  F  P  R  K  V
 8590 ACACATGTGGAAAATGCAACTATTTTTCAGACAGAAAAAACAATTATGTTCAGCATGTTAGAACTCATAC
      Y  T  C  G  K  C  N  Y  F  S  D  R  K  N  N  Y  V  Q  H  V  R  T  H  T
 8660 AGGTAAGAGAAGCTTTCTAGTCCATAAGTTCAGTTCTCTTTTCTGATTGTTACTTGAGTGGTTAGTTAAG

 8730 TAGTGCTTGAGAAAAAGGACTCTGGTTCAAATCCAGGTTCCATTTTGCTATCTTTGTGACCTTGCACAAG
 8800 TTGTTTAACCTCTTTGTTCAGAAATTTCTCCATGGAGTAACAATATCTAGGTTGGGAGGATTATGTGAAG
 8870 TTACATGTAAAGCACAGAGGAACAGCCAAGAGATCTTACCGTGGTCTTACTAAAGTACATATCCTAACTT
 8940 GGGGTTTACCTTCAGCAAGTTAGACTGGCCGTGCATGGTGGCTCACGCCTGTAATCCCAGCACCTCGGGA
 9010 GGCCGAGGCGTAAGGACTGCCTGAGCCCAGAAGTTCTAGACCAGTCTGGGAAACGTGGAAAGACCCCCGT
 9080 CTCAAAAGAAAACAGAAAAAAAAAAAGTTTGTGTATTTATTTCTGTGACAGTGAACACATTTAAGATGCA
 9150 GTGTAGTTTCTGAAAGAAATTGAGGGCTTATATAAAAAGGAAAACTCAGTGATTAACCTTGCACATGCCC
 9220 ACTTAACAAAAGCCTCAAACTTTAAAACATTTGACTTATAGCATTGAGTTCCTAAATGGAACAACTTGTC
 9290 TTTGTCCACAAAAAAGAAATGTGAGAAGGTAGTTTTTAAAAGGTGGGTGGGATTATCACCAATTGAAACT
 9360 ATTTTCCTATGAAAGCAAACACTTGTAGAAAATGTATTTTTCAGACAATTATGTTTAGGGTGTTTGAACT
 9430 GATACTAAGCGCAACTGTATAAAAAAAGTTGAAACTTTTTTTTTTTTTTTTTTTTTGAGACAGTCTGGCT
 9500 TTGTCGCCCAGGCTGGAGTGCAGTGGCAAGATCTCAGCTCAGTGTAACCTCTGCCTCAGGGTTCAAGTGA
 9570 TTATCTTGCTTCAGCCTCCCTGGCTCAGCCTCCCGAGTAGCTGGGATTACAGGCACCCACCACCGTGCCT
 9640 GGCTAATTTTTTTTTTTTGAGACGGAGTCTGGCTCTGTTGTCGAGGCTGGAGTGCAGTGGCATGGTCTTG
 9710 GCTCACTGCAACCTCTGTCTCCCAGGTTCAAGCAGTTCTCCTGCCTCAGCCTCCTGAGTGGCTGGGACTA
 9780 TAGGTGCCCACCACCACACCCAGCTGATTTTGTATTTTTAGTAGAGATGGGGTTTCACCATGTTGCCCAG
 9850 GCTGGTCTTGAACTCCTGACCTCGTGATCCGCCCACCTCAGCCTCCCAGAATGCTGGGATTACAGGCGTG
 9920 AGTGCCACCCCTGGCTAATTTTTGTATTTTTAGTAGAGATGGGGTTTTGCCATATTGGCCAGGCTGGTCT
 9990 CAAACTCCTGACCTCAGGTGATCTACCCACTTTGGCCTCCTAAATTGCTGGGATTATAGGGGTGGGCTAC
10060 CGCACCCAGCCGAAACTTTTTTTTTTTTTTTAATTTCTTTTTGTGGAGATGGGGGGTCTCACTATGTTGC
10130 CCAGGCTGGTCTTGAACACCTGGCCTCAAGTGATCCTCCTGCCACAGTCTTCCCAAGTGTTGGGATTATA
10200 GGCGTGAGCCACTGTGCATACTAGTCATTCACTTTTAAATTGGGTTTTATTCAGCTGCTGTTCACTGTAG
10270 TTTTTTTAATTGGATTTATTTATTCATTTATTGATTTATTTGAGATGGAGTCTCACCCTGGAGCCCAGGC
10340 AGTGGTGTGATCACAGCTCACCACAGCCTCTACCTCCTGGGTTCAGGCAGTCCTCCCATCTCAACCTCCA
10410 AAGTAACTGGGACTACAGATGTGCCCTACCACACCCTAATTTTTTAAAATTTAAATTGGGTTTTAAATAT
10480 TGTAATTGAGGGGCCTCATTTTTCAAATAAATGTTTTTAACTATTGGTAGCTCATTTGGACATTTCTGTT
10550 ACATTTTCTTGGTAAGAATTGGTACATTTGGGTCCTGATGAATGGATAGAGAATAATCCAGTACTCTACT
10620 CTTGGGGTCACAAATGAATAGGGGGTGTGGGATGCTGTTTGCCTCAGGTCTCCTCTTACAGGAGTTGGCA
10690 CTTGACAGGACTGAACCCCACTGGTAAAGGGGACACTGAGGAGGGGAATTGATGCTGCTTTAAGTGGAGT
10760 GGTAGACTTAATTTTTGTTTTGTTTTGTTTTGGAGATAGGATCTCCCTCTGTGCCCCAGGCTGGAGTGCA
10830 GTGACGCGATCTCGGCTCACTACAACCTCCACCTCCCAGGTTCAAGCGATTCTCATGCCTCAGCCTCCCC
10900 AGTAGCTGGGATTATAGGTGCCCACCACCATGGCTAGCTAATTTTTTGTTTTTTTAGTAGAGATGGGGTT
10970 TCACTATGTTGGCCAGGCTAGTCTCGAACTCCTGACCTCGTGATCTGCCTCGGCCTCCCAAAGTGCTGGG
11040 ATTACAGGCGTGAGCCACCGCGCCTGGCCAGTTTTTTTTTTTGAGATAGTTTACTTTGTCACCCATCCTG
11110 GAGTGCAGTGGCGAGATCACTGCTCACTGCAGCCTTGACCTCCTGGACTTGTGACCCTCTCGCCTCAGTC
```

11180 CCCCAGGTAGCTGGGACCACAGACGCATGCCACCACACCTGGCTGATATTTGTATGGTAGAGACAGGGTT
11250 TCGCCATGTTGCCCAGATTGGTCTGTAACTCCTGAGCTCAAGTAATCTGCCCTTAGCCTCCCAAAGTGCT
11320 GGGATTATGGGTGTGAGCCACCGTATCCAGCGACTTAATTTTAAAAATTTATTTATTTACTTATTTTT
11390 TATATCCCAGATACTTAAAACTATTAACTGGCAGGCTTAAACAATAAAACTTGCAGGTGAAATTCCATTA
11460 CAAGAGTGACCTCTGTTACAGGATTGCTAAGCCCCAGCAGATGGATGGCTCATGTGTTGAATGCTTTAAA
11530 ATTTAGTCCAGTAAAAGAATTTGTAGTCCCTTTGGAATTTGTCATAAGCTGTGATTGATTGTCTCGATGC
11600 TTTGAAACCATTCGATTTCAGAAGTAAAGGTCAAGCATTACAAAAATACATAAAGTAGGAAATGAAAGTT
11670 ACTTGGGGACACAACCCCATTAATCACTTGACAGTTTGAATTAGACAGACTGTTAGACTAAACCAGCCTT
11740 ACCTACAATATCTTAAATCCCAGCCATCATATATACTAAACTCATAAATAAGTACACCATATTCATTTAG
11810 CTGGGATTACAGGTGCCTTCCACCATGCCCGGCTAATTTTTGTATTTTCAGTAGAGATGTGGTTTCACCG
11880 TGTTGGCCAGGCTAGTCTTGAACTCCTGACCTCAGGTCGTCTGCCCACCTCAGCCTCCTGAAGTGCTGGG
11950 ATTACAGGCCTGAGCCACCAAGCCTGGCCTGGTTTATTTGGTCTTAATGCTTATTTTTCCCCAGTATTAC
12020 CTTCATTCTGATTTTTTTTTTTTTTTAACTTTTTTTTTTTTTTTTTTTTTTGAGACAGTTTTACTCTTGTCG
12090 CCCAGGGTGGAGTGCAATGGTGCAATCTCGGCTCACTGCAACCTCCGCCTCCCAGGTACAAGCAATTATC
12160 CTGCCTCAGCCTCCTGAGTAGCTGGGATTCCAGGCATGCGCCACCACACCTGCTAATTTTGTATTTTTAG
12230 TAGAGACAAGGTTTCTCCATGTTGGGCAGGCTGGTCTCGAACTCCCGGCCTCAAGTGATCCACCCGCCGC
12300 GGCCTCCCAAAGATTACAGGCGTGAGCCACCACGCCTGGCCAATTTTGTGTGTGTGTGTGTGTGTATTTT
12370 TTTTTTTTTTTTTTTTTTTTGAGATGGAGTCTTGCTCTGTTGCTCAGGCTGGAGTGCAATGGCGTGATCTC
12440 TGTTCACTGCAACCTCTGCCTCCAGGGCTTAAGTGATTCTCCTGCCTCAGCCTCCCGAGGAGCTGGGATT
12510 ACAGGTGCGTGCCACCACGCCCAGCTAATTTTTTAAAAAATTTTTAGTAGAGACAGCGTTTCACCATGTT
12580 GGCCAGGCTGGTCTCGACCTCCTGACCTTGTGATCAGCTGACCTCGGCCTCTCAAGGTGCTGGAATTACA
12650 AGCGTGAGCCACCAAACCTGGCCTTTATTACATTTTAAAACAATTTGGTGATGTATAGATTGAAGGTTAA
12720 TATTTTGATTATGTAAATTCTGAAAGTATCTCTGGTATTAAATGGATAGTTCTGAAAATTATGGAAAATA
12790 AACTCATCTGGTGCGGAAGAGTAGAGACAGAAAAGACAGGAATCACTTAAATTGATAGTGTTCAGGAATT
12860 ACTTCTCATTTCCACTTGCATTAGTAAGTAGCCCAGTTTTCTGATTGGCTTAGTGGGCTTAATTGTGTTT
12930 CTTGTGCCCTTCTATTCTGTGCTAATCTTCAGAACAATCCATTGAGGAAAATAGCATCTGTGATGATAGT
13000 TCATGTTTACACAGGGGAAAACTTCAGATTTAAAAGGCTGAAGTGATGTTCCCAAGGTCATATACAAGTA
13070 TCATTTATAGCCGTTCCACGATGGCTCACGCCTGTAATCCTAGCACTTTGGGAGGATGAGGCAGGAGAAT
13140 CGCTTGAACCTGGGAGGTGGAGGTTGCAGTGAGTCGAGATTGTGCAACTGCACTCCAGCCTGGGGGACAG
13210 AGTGAGACTCTGTCTCAAAAACAAACTACAAGTAGCATTTAAAGTTCAGCAATCTCGACTCACTGCAACC
13280 TCCACCAAAAGGGTGGGGGGAGCTGGGGACTTTCATTCTGGTTGACCTCTTTAGTTTAGTGGTTTTGATA
13350 TTTAACTTGTTCACAAAATCTACTTTTAAAGAAAACCTTTTTGCCTGTATGTGTAACTGAAAAATCAGTA
13420 GGTAACTAAGCTTTCTGGAGGAGAGTTATGCCTAGTTTGTGGAATTACACAGATAAAGTAATCTGTTGTA
13490 AAGAATTTAAAAGCAGGCTGGGCGAGATGGCTCATGCCTGTAATCCCAGCACCTTGGGAGGCAGGCAGAT
13560 CACCTGAGGTTGGGAGTTTGAGACCAGCCTGGTCAGCATGGTGAAACCCTGTCTCTACTAAAAATACAAC
13630 ACTTAGCCTGTAGTCCCAGCTTCTCGGGAGGCTGAGGCAGGAGAATCACTTGAACCCAAGAGGCAGAGGT
13700 TGCAGTGAGCCAAAATCGCGCCACTGCACTCCAGCCTGGGCGACAGAGCGAGACTGCGTCTCAAAAAAAA
13770 ATAATAATAAAATAAAAGAATTTAAAAGTAAATTATCTTTGATTCTTCAAGTTTATCTGCAGTGGTATTT
13840 AATTTTTTATTGAGATACAGTTTACATATGCAGTTCACTATATATTGGCCTGTTCTGAATATTTCACACA
13910 AGTGGAATCATAATAAGCAGTCTGAATAATGCTGCTATGAATATTCATGTTTAAGTTTTGTGTGGACGTA
13980 TGTTTTTGTTTCTTTTGGATATATACCTAAGCGTGAGATTGTGGGGCAGTATAGTAGCAACATTTTGGGT
14050 AATTGCCAAACTGTTTTCCAAATTAGCCATGCCATTTTACAATCCCACCAGCAATGTGTGAGGGTTCCAA
14120 TTTCAGCACATCCTTAGCAATAATACTGTTAACATCTGTCTTTGTGATTACAGCTATCTTAGTGGATATG
14190 AAGTCTTCATCTCCTTGTGGTTTTGATTTGCATTTCCCTCACTGCTAATGATAATGTTCATCTTTTCATG
14260 TGTTTATTGGCTATTTGTAAATCTTTTTTTTACAGAATTCCATTAACATAAAACTATCTTAATTGTTCCA
14330 GGTCATAAGGTAGGCATTCTGTTGCTACCAACTCTTTACAGCTAATTCGTAGCTACCAGCGAATTACAGC
14400 AGAATAGTTATGAGGAACACATTTCCCATCTATCACCATTCTTTTTGTTGTTGTTTGTTTTTTGTTTTAA
14470 GACAGGGTCTCGCTCTGTTGCCCTGTGTGGAGTGCAGTGGTGTGGTCACGGCTCACTGAAGCCTCGGCCT
14540 TCTGGGCTCAAATGATCCGCCTACCTCCCAAAGTGCTGGGATTACAGGCATGAGCCACCATGCCTGGCCT
14610 GTTCTTTTTTCTGTGTACCACCTTTTCCTTATTTTATTAGACACATTTGTTTCTGTCTTAGACAAAATTG
14680 ATAGCTTACATTTATTGAGGGCTTTACTATGTGCCAGACACAGTGTTAAATGCTCTGCATGTATTATTTA
14750 AGCTTTTGCAATTACAGGAAATGAAGATTCTGTTACTTTTACAGATGGGGAAACGGTGTTTTAGTAGCTT
14820 TACCAAGGTCATAATTATCTTGTGTTTGGAGACTATATTAAAACAAATTGTATACCTTTTTTTAAAAGCT
14890 TGTTTTGCCGGGTGTGGCGGCTCATGCCTGTAATCCCAGCACTTTGGGAGGCCGAGGTGAGTGGATCACG
14960 AGGTCAGGAGATCGAGACCATCCTGGCTAACAAGGTGAAACCCCGTCTCTGCTAAAAATACCAAAAAATT
15030 AGCCGGGCATGGTGGCGGGCGCCTGTAGTCCCAGCTACTCAGGAGGCTGAGGCAGGAGAATGGTGTGAAC
15100 CCAGGAGGCATAGCTTGCAGTGAGCCGAGATCTCACCAGTGCACTCCAGCATGGGCAACAGAGTGAGACT
15170 CCGTCTCAAAAAAAAAAAAAAAAAAGGCTTGTTTTGTTGTTGACAGCCATGTCTAAAAAGAATATACGTTT
15240 CCTACCTTTGAGTCTTAAATTCTTCAGTATTAAATCACTGGCCTCTAGTCTTCAGTCCCTGTGGCTTTTA
15310 TAATTTGTTTTAAAAATTGATGACTGGTTGGGCATGGTGGCTCATGCCCGTAATCCCAGCACTTTGGGAG
15380 GCCAATATAGGCGGATTGCTTGAGCCCAGAAGTTCGAGACCAGTCTGGGCAACATGGCAAAACCTGGTCT
15450 CTACCCAAAAAAAAAAAAAAATTAGCTGGGCTTGGTGGCACACGCCTATGGTCCCAGCTACTTAGGAGGCT
15520 GAAGTGGGAGGATCTGTTGAGCCCGGGAGGTCCAGGCTGCAGAAGCTACGTTCATGCCACCACACTCCAG
15590 TCTGGGCAACAGAGCGAGACCCTGTCTCATAAAAAACAATAAAAAGAAAATTGATGACCTTCTCTTAAAT
15660 TTTTCTTTTTAGCTTAATGTTTTTTATTTATTTTTATTTTTAGAGACAGGCTCTCTCTCTGTACTCCAGG
15730 CTGGAGTATAGTGGTGCCGTCATAGCTCATTGCAGCCTCAAACTCTGGGCTCAAGTGATCCTCCCGCGTC
15800 AGCCTCCCAAGTGAGACTACAGGGTCTCACTTTCACCCAGGCTGGAGCACGGTGGTACAATCATGGCTCA
15870 CACCAGCCTCATCCTTCTTGGGCTCAAGCAATCCTCCCATCTCAGCCTCCCAAGTAGCTGGGACCACAGG
15940 CATGCGCCACCACGCCTGGCTAATTTTTCTGTTTTTTGTAGAGAGGAGGTCTTGCTATGTTGCCCAGGCT
16010 AGTCTTGAACTCCTGGGCTCAAATGATTGGCCTGTCTTGGCCTCCCAAAGTGTTGGGGTTACAGTCAGGG
16080 GCCACAATCCCCATGCTCAGCCCAGGCTATTTTTTTTTTTTTTTTTTAAACGAGATGAAGTCTCACTCAGCC
16150 TCCCGAGTAGCTGGGACAACAGGCACGTACCACCATGCCCGGCTAATTTTTTTTGTATTTTTAGCAGAGA
16220 TGGGGTTTAATTATGTTGGCCAGGCTGGTCTTGAACTCCTTACCTCGTGATCTACCAGCCTCAGCCTCCC
16290 AAAGTGCTGGGATTTCAGGCGTGGGCCACCGTGCCCAGCCCCAGGCTAGTTTTTAAAATAAAATTTTAGA
16360 AATGAGGTATTGCTGTGTTGCCCAGGCTGGTCTGGAACTCTACCACTTGAAAATTTTTAGCATAAAGCAA
16430 TTACATTCTCACTCAGAAGTATTGTTTTAAGCTAACACTTTCAAATGGTAGTAGATTCATCATTTACAAG
16500 TTAAAAAAAAAACCTAAAAACCGGTGTTAATAGCATCAGACAGTTCAGGTAGATTTTTTAAAAGGTGATT
16570 CTTAATATAACAAAAATGCTTTGTACCTTATCAAGATAGTTTTTTACTTATCACTACTTAATGTATTAAC
16640 ATGCAGTATCACTGTGAAAATTAAAAGGGCTGTGAGAATTCGGAAAGTCAATGTTGTTTTCTTGTCAGCA
16710 AAGCCAAAATAATGTAGTCATAGTTTCTGAAATAAATAACTTACATTTTAAGTACATGATAAATTGATCT

```
16780 TTTTGCCAGAAATAGGAAAAAAAAATTCTTGTTAAAATGTGCTGAGCGCTGAACATTGTTGCATTGTTAG
16850 TGAGAATTGTATTTGGCTATTTCGTGACATTTAAACACTCTTATATTATTGAAATTTTGCAGGAGAACGC
                                                                    G  E  R
16920 CCATATAAATGTGAACTTTGTCCTTACTCAAGTTCTCAGAAGACTCATCTAACTAGACATATGCGTACTC
       P  Y  K  C  E  L  C  P  Y  S  S  S  Q  K  T  H  L  T  R  H  M  R  T
16990 ATTCAGGTTGGTAAGAAATTGGAACTTTTCTATCATTTTCAGTAAATGACCATTTTAAGGAAATTCTTTT
       H  S
17060 AGACTTGTAACCGAGTCATTTACTTATTATTTATTTTTAATATAATTTAGAAGTCAGAATTTAAAAATCA
17130 GTATTCCGTCAATCTGGGGATTCCTAACATGGAATCCAAAGGTGGACCTCCTGAAAATGGAATTTTTGTG
17200 TACACTTTTTCTGGGAGGAGGGGCCTTAATTCTCATCAAATCTTTGAAGCGTTCCGTGACTCCAACAAAA
17270 GTTAAACTAATGTAGACCAAGACTGATTGATCTTTTAACCCCTACTTCTCATATTTCTCCTAACAGTATA
17340 AAGCTGAGAGTCACAACGTGTGGTTGACAAGTGTCAGGGATTCCTTCTTAGCTTCTCAGGTCCTGAAATG
17410 AAGGTTTGTTCTGTTTCAAGGTTTGGTCGAGCTTTTGAGGACTGACTGCAAAAGTTTAGCTTGAAAAAGA
17480 AAATGATAGACAATCTAGGATGCCAATTTATTAAGCTGTATTAAGAGTATAGGACTGTAACATGAGAGAT
17550 TCGTCCACTCCAGTGACTGAAACAATCACCATCACCACTCATAATTAGTCCCTGTTGGCCAGCATGGTGG
17620 CTCACGCCCTTAATCCCAGCACTTTGGGAGGCCAAGGTGGGCGGATCACCTGAGGCGGGGTAGTTTGAGA
17690 CTAGCCTGACCAACATGGAGAAATCCTGTCTCTACTAAAAATACAAAATTAGCCAGGCGTGGTGGCGCAT
17760 GCCTGTAATCCCAGCTGCTCAGTAGGCTGAGGCAAGAGAATTGCTTGAACTTTAGGAGGCAGAGGTTGTA
17830 AGCCGAGATCACGCCATCAAGAGCGAAACTCTATCTTGAAAAAAAAGTCCCTATTGTCAAAGTATTCAAG
17900 TGGCCAAGTTAAAAAGTAATGTTAGCATTGGTGGTCTTGCTTGAATGGGTAGCCCTGATTGACTCTTGGT
17970 GAACTACCACTTTTACTTTTTATTTTTATATTTTTTTTGGAGACGGAGTCTTGCTCTGTCGTCTAGGCTG
18040 GAGTGCAGTAGTGCGATCTCAGCTCACCGCAACCTCCGCTTCCCAGGTTCAAGCGATTCTCCTGCCTCAG
18110 CCTCCTGAATAGCTGGGATTACAGGCGCGCACCACCATGCCCGGCTGATTCTTGTATTTTTAGTAGAGAT
18180 GGGGTTTCACCATGTTGGTCAGGCTGGTCTCGAACTCCTGACCTCATGATCCACCCACTTTGGCCTCCCA
18250 AAGTACTGGGATTACAGTCATGAGCCACCATGCCTGGCCGAACTACCACTTCTAATTAGAATACAGATTT
18320 AGAATGTATATCCCTGTGGGAAAAGTATCTTAAATCTTGCTGTTACAAAAGCCTATGTAGAACCATCTCC
18390 CTTAAGTTAATAATTAGCTTAGATCTGTGCTATTCGCTTGAGTAGCCACTAGCCACATGTGGCTATTAAG
18460 CTTTCGAAACATTAGTAGTTTGAATTGATACTTATAAAATATGTACTTACCAGATTTTTTAAATTTTATT
18530 TTATTATTATTTTTTTTCTGTTGAGACAGAGTCTTGCTGTATCCCCCAGGCTGGAGTGCAGTGGCGCGCA
18600 TCTCAGCTCACTGCAACCTCTGCCTCCGGGGTTCAAGCAATTCTCATGCCTCAGCCTCCCCAGTAGCTGG
18670 GATTACAGGCATGTGCCACCATACCCAGCTAATTTTTGTAATTTTAGTAGAGATGGGGTTTCGCCATGTT
18740 GGTCAGCTTGGTCTCGAACTCCTGGCCTAAAGTGGTCTGCGCATCTTGGCCTCCCAAAGTGCTGGGATTA
18810 CAGGTGTGAGCCACCGCGCCCGGCCACAGATTTCAAACAATGTGAAGAAAAGACTATCTTTCTCTGAAGC
18880 AGGTTAGAGAAAAAATAAAAGAATGTTAAATATTTATTTTTCAAGTTGATCATATGGCAAAATTATTGTA
18950 TACATTGGCTTTAAAAAGTAATAAAATTAATTTTAACTGTTTCCTTTTTACCTTTGTGGTATGACTACTA
19020 GAAAATTTAAGATTACATGTGTGATATGCATTATATTTCTACTGGACATTGCTGCCATGAGTTAGTAACA
19090 AGACTTGATAATAAGACTTGATTATGTCACAGATTCTGATTTATTTGAACAGCTATGAAGGTGTCCTGTA
19160 GTATAGTTGCTAGCCTTTCTTGACCCTACGTGCTTGTCACGCTAATGTACTTTGAGTGTTTTTTTCCTAA
19230 ATCTAAGCCATTGAGTGGTGCCACTTAACCAGAAACCTGGTTTTATTATTTTTGGGTGTTTTTTGGTTTC
19300 ATTCTCTGATGGTAATGTGGATGCAATTTCAGACTTTGGAGGTAAGCATCATGTCCTGTTGATAGTTGTA
19370 CTAGCTTTTTAGTAAAATATTTGAGAGCTTTGTATTATTATTATTATTATTATTATTATTATTATTATTA
19440 CTTTTTTTTTTTTTCTGAGATGGAGTTTCGCTCTTGTTGCCCAGGCTGGAATAAAATGGTGTGATCGCAG
19510 CTCACTGCAACCTCCGTCTCCCAGGTTCAAGCGATTCTCCTGCCTCAACCTCCCAAGTAGCTGGGATTAC
19580 AGGCATGTGCCACCACACCCAGCTAATTTTGTATTTTTAGTAGAGATGAGGTTTCACCATGTTGACCAGC
19650 CTGGTCTCGAACTCCTGATGTCAGGTGATCCGCCTGCCTCAGCCTCCCAACGTGCTGGGATTACAGGCGT
19720 GAGCCACTGCTCCCGGCCAAGAGCTTTATATTAGTTAACTAGTTTTTGTCTTCATAAATATTTTATGTTG
19790 CATGAGTTCTATTATCCCCATTTTACAGTTGGGGAATGTAGGGCATTGAAAAAAATACGACCTTTTTTCC
19860 TGTAGAATGTCCTTCCTTCTGGATATGTCCACTTGTTTTCTCATGGTGTCATTTAGCTTTTCTTCTGTCC
19930 TTTGTATTGTAAACTGGAAATTAAATTTAAATGCTTGATAATCTTTCTCGCAAATGATATGCCTCATTTA
20000 AAGTCACGTGGTTGACCCATTAATATAATGTTAGGTCACTGCATTTAGAATGACTACATACTTTTATCCT
20070 TTCGTTCAATTAGTAATACTTTGTCTTGTCACTGTTTAAATGTTTATGGTGTTTTACACCAGTTGATAAT
20140 CTTTGACTGAAACAGTTGTATTTGGGTTTGCAAAATGTTTTGAGTTTTGCCTGCTGTATTTTAAAGATGA
20210 TTTTTAGTATTTGCTGTTACAGTATAGCAAGTATTTTAATGGTGTCTTAAAAACTAGTAGTAGCCACTAG
20280 CTATTGTATGTATGCTGAATAGTAAAAACAAGTATTTGCTGTCTCCAGTCTTAGTACTACGAAGATACTG
20350 AGTTTCAGATTACATATTATAGGATACAAGCTCACAAATAAGTAATTTTGTTGTTGTTGTTTTTGGAGTT
20420 GGGGACTCTGCTGCGCAGGCTGGAATGCAGTGATGCGATCATGCCTCACTGCAACTTCATATGCCTAGGC
20490 TCAAGCCATCCTCTTGCCTCAGTCTCCTGAGTCGCTAGGTCTACTGGCACATGCCACCACTCCAGCTAAT
20560 TGGGGACTATTATTATTATTATTATTTTTGAGACAGTTTCGCTCTTGTCACCCAGCTAGAGTACAATGGT
20630 GCGATCTCGGCTCACTGCAACTTCTACCTCCCGGGTTCAAGCTATAGAATCTTCCTCAGCCTCCTGTGTA
20700 GCTGAAATTACAGGCACCCGCCACCATGCCTGGCTGATTTTTGTATTTTTAGTAGAGGTGTTGATCCACC
20770 CACCTCAGCCTCCCAAAGTGCTGGAATTACAGGCATGAGTCACCGCGCCTGGCTGGAACTATTATTTTTA
20840 TTTAAATATCGTATTTATTTATTTATTGAGACTGAGTCTTGCTGTCGCCCAGGCTGGAGTGCAATGGTGC
20910 GACCTCGGCTCACTGCAACCTCCACCTCCTAGGTTCAAGCGATTCTCCTGCCTCAGCCTCCCTAGTATCT
20980 GGGACTACAGGCTCGTGCCACCATGCCAGGCTAATTTTTTGTATTTTTAGTAGAGACGGGGTTTCACCAT
21050 GTTAGCCAGGATAGTCTTGATCTCCTGACCTCATGATCTGCCCGCCTCAGCCTCCCAAAGTGCTGGGATT
21120 ACAGGCGTGAGCCACCACACCCGGCCAATATCCTATTTCTTTCTATGGAGACTGGGTCTTGCTGTGTTGC
21190 CCAGGCTGGCCTCAAACTCCTGGCCTCAAGCAGTTCTCTTGTCTCAGCCTCCCAAAGTGTTGGGATTACA
21260 GGTTTGAGTCACTGTGCCCAGACAGACATGTTTTATTTTTTTCTGTTGATAACGTTGGTGCCAAGGGACT
21330 TTTAAATAGCTGGGATTGTTTGAAAGATATTGTGATGGCTTGAGAGTCTTTGTAGAATAAATTTTAAAAA
21400 CTATTTTATTGAGATATAATTTATAAATTTAAAAATGCACAGGTCTTGAGTGTACAGCTCAGTGACTATG
21470 CTTTCACCTATTTGATTATCACTCAAGAACAAGAGATGGAATATTTCTGCTCAGAAACACCTTGCCCTTT
21540 CCACCCAGTCTTTCCCCTGGATGATATTCTGTCTCCTACCAAAGAACATTATAGAATTTGTTTGTTTGAG
21610 GTGGGATCTCACTCTGTCACTCAGGGTGGAGTGCAGTGGGTGCAGTTACAGCTGTCTAAACCTCTTGGGC
21680 TCAGGCAGTCCTGCCACCTTAACCTCCCAAGTAGCTGGGACCACAGGTGTGCGCTACCATGCCTGTTTTT
21750 TTTTTTTCTTTCTTTTTTGTAGAGATGGAGTCTTACTATGTTGGCCCAGGCTGGTTTTGAACTCCTGGGC
21820 TCAAGCGATCCACTTGCCTCACCCTACCAAAGTGCTAGGAGTACAGGCATGAGCCACTGCACCTAGTTTT
21890 AGAACATTCTTTTATAAAATGTTCTTGAGATTTATCCATGTTGCGTTATTGGACTTCATTTTATTGCTGG
21960 TAATGTATTGTATGCCGCCATTACTTTTTACTTAAAAATTTTCTTTGCCGGCTATGCACGGTTGTTCACA
22030 CCTGTAATCCTAGCGCTTTGGGAAACCATGGTGAGCGGATCATCGGAGGTCAGAAGTTCAAGACCAGGCT
22100 GGCCAACATGGTGGTACCCCATCTCTGCTAAAATACAAAAATTAGCCTGGCATGATGGCGGGTGCCTGTA
```

22170 ATCCCAGCTACTCCGGAGGCTGAGACAGGAGAATCACTTGAACCCGGGAGACGGTGGTTGCAGTGAGCCG
22240 AGATTGCGCCATTGCACTCCAGCCTGGGCAGCTGAGCGAGACTCAGTCTCAAAAAAAAAAAAAAAAAAAA
22310 TTTCTTTGCCATTCTCTCTTGGTTTTTGTTTTGTTTTGTTTTTATTTTTAAGGGAATTAATTTAAAAACT
22380 TAAATTGAGAGGCTTGTATTTGTGTATTTTTCACTGCGGCTTTAACCTCCTAGGCTCAAACGATCCTCCC
22450 ACCTCAGCCTCTGGAGTAGGTGGCACTGTAGGCTTGTGCTATAACATGTGGCTAATTTAAAAATTTTTTT
22520 GTACACAGGGTCTCACTAGGTTGCCCAGACTGGTCTCAACCTCCTTAGCTCAAGCAATCTTCCTCACCTC
22590 ATCCTCCCAAAAGTGCTAGGATTACGGGTGTGAACCACTATGCCTGGCCATTCTTCTAAGAATATTTTTG
22660 ATACGTATGTGTAAAGCTTGCAGAACTCTTTCCCCTTTTGTCTTGGCTTACACCTATAAATTCATTTCGG
22730 AGGCATAAAACAGACAATTGGTAGATTTATAAAGAAGAGAACCTGTTACTAGAATTGGGAAAAAGAATTT
22800 AAAATTGTTTGTACAGGGCAAAAGTTAATATTCAGGTAGGTACTAATTAGAAAATCTTGATGAATATATT
22870 TTAATATAAGCAAGACTTCATGGCAATAAAGTAAAAATTACGGGAGAAAAAGAACGGAGCAAATAAATCA
22940 TGCCATCAAATTTTAAACAGAAAATTTCTACATCCTCCCTTTCCGTTCACCATTCCTTAGGTGGACCCTC
23010 GCACCTTGATGGATGCATGCTTGCTCTATAGATCTCCTTATTCATCAGAGTTATAAAATGGTGCTATTTT
23080 AATTATAGTTATTTTATTAGTGTGTAAATATGAAGAGAAGAGATAAATTCCACTTATCTATTTACTCATG
23150 ATGTAGGAAAGACAGGATACGTAAGAGTCTTCCCCTTGATTGATCGATTGATTGAGATGGAGTCTCACCC
23220 TGTCGTCCAGGCTGGAGTGCAATGGCACGATTTCAGCTCACTGTAACCTCCGCCTCCTGGGTTCAAGTGA
23290 TTCTCCTGCCTCAGCCTCCCGAGTAGCTGGGATTACAGGCATGCACCACCACGCCTGGCTAAGTTTTTGT
23360 ATCTTTAGTAGAGATGGGGTTTCACCATATTGGCCAGACTGGTCTCCGGCTCCTGGTCTCCAACTCCTGA
23430 CCTCTTGATTCGCCTGCCTCGGCTTCCCAAAGTGCTGGGATTACAGGCGTGAGCCACTGCGCCCAGCCTT
23500 TTTTATTTATTTATTTTTTTGAGATGGAGTCTTGCTCTGTTGCCCAGGCTGGAGTGCAGTAGTGCGATCT
23570 CGGCTCACTGCACCCTCTGCCTCCCGGGTTCAAGGGATTCTCCTGCCTCAGCCTCCCAAGTAGCTGGGAC
23640 TGTAGGCACGCATCACCACGCCTAGCTAATTTTTTTGTATTTTTACAAAATGCCTCCCCAAATTTTATAT
23710 TTTTACAAAAATGCCTCCCAAAGTTTTGGGACTACAGGCGTGACCCACCGCGCCCTGCTGAGTCTTCCCT
23780 TTTATTTATCCATACTCAAAGTTACCAATTGGTTCCAAAAGTGAACAATTATAGGTAACACATTTTGAGT
23850 TCTTGCTGTCTGCTCCATGTTAAGGACTTTACATAGATTAGATTATTTATAGCTCACAACACTTAGAGAG
23920 TATTTTATAGATGAAATTGAGGCCGGGCGCGGTGGCTCACGCCTGTAATCCCAGCACTTTGGGAGGCCGA
23990 GGAGGGTGGATCACCTGAGATCAGGAGTTCGAGACCAGCCTGATCAACATGGAGAAATGCCGTCTCTACT
24060 AAAAATACAAAATTAGCCAGGCTTAGTGGCACATGCCTGTAATCCCAGCTACTAGGGAAGGTGAGGCAGG
24130 AGAATCGCTTGAACCTGGGAGGCGGGGGTTGCAGTGAGCCGAGATCGCGCCATTGCACTCCAGACTGGGC
24200 AACAAGAGTGAAACTCCATCTCAAGAAAAAAAAAAAAAGAAAGAAATTGAACTCTAGAGAGGTTTCAGGAC
24270 TTTGCTCAAAGTCTCAGAGTGAGCAACTTGCAGAGCTCATGTTTTATCTCCCAGATGGTCTAGCTCTATC
24340 TAGAAGTTCTTAGGCCTTATGCTACCTATCTTTCTACATTCTATGTCCACGTATGTTAAAATGTTTGTCT
24410 CCATTTAAATCATTCACTGGAGAGAACCAGGTATAGAGTAATGTAGCATGTTGTAGAAACTTGCATATTT
24480 GTAAATCTGTTAGTAGACCAACATGGAGAAGAATTGTCCAGTCAGCTGAATATCTAATTTGACTACCTGG
24550 ATAAGGTGATTATTTTATCACCAGGCATTTTCTAATCATGTAAAACTTAACATCTACATGGTTTTACCTA
24620 ATGTCTGAGTTTTGCTTTATGAAAATTTTTAGTACAATTTGATATGAATTTGAGAATGTTTGAGATGAAT
24690 TTTCATAAATGTCAATTTCCGTTTTTCTACTATGCATTCCATTGGACCAGTGGGGTATGGATACCATTTG
24760 GTAATATTTACTAGAGTGTGATCTAGATGGGTATGTATTCAGGTAGAATGTATTACTCTTATGCCTTTAA
24830 TTTGGGGGTGGGGGTTCTGGTTTTTTATGTATCAGTGATTGTGACTTTGCTTATTTTACATACCATATCT
24900 ATTTGTGTGCCACTTCTCATTACCTAGTTCCTTTATGTATTTTATAGAAAGGTGGGGGGAAAATCAAGAA
24970 AGTTTTCTGAATAATTACCTTGGCCTATATTTTTGCTTTAAAATAGCAATGCTAGCGCAGGAAGTTTGCT
25040 AAGTGAAAATTTACTCAGCTCTTGCCATGACGGACAGAAGGATTAAATTTCAAGAAAAAGCCTTTGCTCA
25110 GACTAAAGCAGAATTTCAAAACTTTTGCACCTAGGATCTGGGAGTATCGCCCAGAGTGAAAGATATTTTT
25180 CTCAAGATTTACCATAAGAAATTTAGGGTGGAGGGAGTATGGGAATTTTATTTTATTTTTATTTTATTTTT
25250 GAGACGAAGTTTCGCTCTTGTCGCCCAGGCTGTAGTGCAGTGGTGCGATCTTGGCTCACTGTAATCTTGC
25320 CTTCTGGGTCCAAGCAATTCTCCTTCTTCAGCCTCCTGAGTAGCTGGGATTACAGGCATCTGCCACTACA
25390 CCCGGCTAATTTTTTGTATTTTTAGTAGAAACAGGGTTTCACCATGTTGGCCAGGCTGGTCTTGAACTCC
25460 TGACCTCAGGTGATCCACTGCCTTCGCCTCCCAAAGTGCTGGGATTACAGGTGTGAGCCACCACACCTGG
25530 CTGGGAATTTTATTTATTGATGTATTTTTGAGACAGGGCCTTGCCCTGTTGCCCAGGTTGGAGTACAGTA
25600 ACACATTCACAACTCCATGCTACTTCAGATTCCTGGGCTTAAGTGGTCCTCCCGCCTTTGCCTCCCAAGT
25670 AGCTGGGACTGCAGGTGCACTCCACCATGCCTGGCTAATTTTTTTTTTTTTTTTCTTTTTGTGAGACAAAG
25740 TCTTACTGTCGCCCAGGCTGAAGTGAAGTGGTGGGATCTCAGCTGACTGCAACCTCCGCCTCCTGAGTTA
25810 AAGCAATTCTCGTGTCTCAGCCTCCCTAGTAGGTGGGATTACTGGCATGCAACACCACGCCCAGCTAAAT
25880 TTTTTTATATTTTTAGTAGAGATGGGGTTTTACTATGTTGGCCAGGCTTGCCTTGAACTCCTGACCTCAG
25950 GTGATCCACCTGCCTTGGCCTCCCAAAGTGCTGGGATTTACAGGTGTGAGCTACCGCACCTGGCTGGGAA
26020 TTTTATGTATTGATTTATTTTTGAGACAGGGCCTTGCTCTGTTGCCCAGGTTGGAGTTCAGTAGCACATT
26090 CACAACTCACTGCTACTTCAAATTCCTGGGCTTAAGCGGTCCTCCCGCCTTTGCCTCCCGAGTAGCTAGG
26160 ACTACAGGTGCACTCCACTATGCCTGGCTAATTTTTTTTTTTTTTTTTTCTTTGAGACACAGTCTCGCTG
26230 TCACCTAGGCTGGAGTGAAGTAGTGCGATCTCAGCTCACTGCAACCTCTGCCTCCTGGGTTAAAGCAATT
26300 CTCGTGTCTCAGCCTCCTGAATAGGTGGGATTACTGGTGTGCAACACCACGCCCAGCTAATTTTTTTTGT
26370 ATTTTTAGTAGAGATGGGGTTTCACCGTGTTGGCCAGGCTGGTTTCAAACTCCTGGCCTCAAGGTATCCG
26440 CCCACCTTGGCCTCCCAAAGTTAATTTTGTTTTGATTTATTTTGCTCAGGTTGTTCTCTAATTCCTGGGC
26510 TCAAGCAGTCCTTCTGCCATGGCCTCCCAGAGAGTTGAGGTTATAGTTGTGAGCCACTGCACCTGGCCAG
26580 AATTTTATTTTCTACTCCATTAGGCATTTAACTAAACACACAAAAAAATTTTTAAGTTGATAATATTCCT
26650 CCAAATTAACATCCAGAGCCCAATTGAACTTTGCCAATATGTGTATGTCTTAAAGAATCTCTGTTCTTTA
26720 GTAGTGCTTGAGGACTCTAACTTGAACTTTTTTGAAGCTTGTGGTTAACTGAGATTTATTCCTCTCTTTT
26790 GAGAATCAATGTCAAGGGATAGAGAAATGTTATACAAATAAATATGGTAAATGTAAGGTGCATGATACAG
26860 CATTTTAAATAGTCTTTGTTGTTACTTTACATATACAGTTCTTTATATTTTAATCTTGAATTTGTCTTAA
26930 TCTATGTCTTCTCTCATACTTTTGATTTGCCTTCAGGTGAGAAGCCATTTAAATGTGATCAGTGCAGTTA
G E K P F K C D Q C S Y
27000 TGTGGCCTCTAATCAACATGAAGTAACCCGCCATGCAAGACAGGTTCACAATGGGCCTAAACCTCTTAAT
V A S N Q H E V T R H A R Q V H N G P K P L N
27070 TGCCCACACTGTGATTACAAAACAGCAGATAGAAGCAACTTCAAAAAACATGTAGAGCTACATGTGAACC
C P H C D Y K T A D R S N F K K H V E L H V N
27140 CACGGCAGTTCAATTGCCCTGTATGTGACTATGCAGCTTCCAAGAAGTGTAATCTACAGTATCACTTCAA
P R Q F N C P V C D Y A A S K K C N L Q Y H F K
27210 ATCTAAGCATCCTACTTGTCCTAATAAAACAATGGATGTCTCAAAAGTGAAACTAAAGAAAACCAAAAAA
S K H P T C P N K T M D V S K V K L K K T K K
27280 CGAGAGGCTGACTTGCCTGATAATATTACCAATGAAAAAACAGAAATAGAACAAACAAAAATAAAGGGG
R E A D L P D N I T N E K T E I E Q T K I K G

```
27350 ATGTGGCTGGAAAGAAAAATGAAAAGTCCGTCAAAGCAGAGAAAAGAGATGTCTCAAAAGAGAAAAAGCC
      D  V  A  G  K  K  N  E  K  S  V  K  A  E  K  R  D  V  S  K  E  K  K  P
27420 TTCTAATAATGTGTCAGTGATCCAGGTGACTACCAGAACTCGAAAATCAGTAACAGAGGTGAAAGAGATG
        S  N  N  V  S  V  I  Q  V  T  T  R  T  R  K  S  V  T  E  V  K  E  M
27490 GATGTGCATACAGGAAGCAATTCAGAAAAATTCAGTAAAACTAAGAAAAGCAAAAGGAAGCTGGAAGTTG
       D  V  H  T  G  S  N  S  E  K  F  S  K  T  K  K  S  K  R  K  L  E  V
27560 ACAGCCATTCTTTACATGGTCCTGTGAATGATGAGGAATCTTCAACAAAAAAGAAAAAGAAGGTAGAAAG
      D  S  H  S  L  H  G  P  V  N  D  E  E  S  S  T  K  K  K  K  K  V  E  S
27630 CAAATCCAAAAATAATAGTCAGGAAGTGCCAAAGGGTGACAGCAAAGTGGAGGAGAATAAAAAGCAAAAT
        K  S  K  N  N  S  Q  E  V  P  K  G  D  S  K  V  E  E  N  K  K  Q  N
27700 ACTTGCATGAAAAAAAGTACAAAGAAGAAAACTCTGAAAAATAAATCAAGTAAGAAAAGCAGTAAGCCTC
        T  C  M  K  K  S  T  K  K  K  T  L  K  N  K  S  S  K  K  S  S  K  P
27770 CTCAGAAGGAACCTGTTGAGAAGGGATCTGCTCAGATGGACCCTCCTCAGATGGGGCCTGCTCCCACAGA
      P  Q  K  E  P  V  E  K  G  S  A  Q  M  D  P  P  Q  M  G  P  A  P  T  E
27840 GGCGGTTCAGAAGGGGCCCGTTCAGGTGGAGCCGCCACCTCCCATGGAGCATGCTCAGATGGAGGGTGCC
        A  V  Q  K  G  P  V  Q  V  E  P  P  P  P  M  E  H  A  Q  M  E  G  A
27910 CAGATACGGCCTGCTCCTGACGAGCCTGTTCAGATGGAGGTGGTTCAGGAGGGGCCTGCTCAGAAGGAGC
       Q  I  R  P  A  P  D  E  P  V  Q  M  E  V  V  Q  E  G  P  A  Q  K  E
27980 TGCTGCCTCCCGTGGAGCCTGCTCAGATGGTGGGTGCCCAAATTGTACTTGCTCACATGGAGCTGCCTCC
      L  L  P  P  V  E  P  A  Q  M  V  G  A  Q  I  V  L  A  H  M  E  L  P  P
28050 TCCCATGGAGACTGCTCAGACGGAGGTTGCCCAAATGGGGCCTGCTCCCATGGAACCTGCTCAGATGGAG
        P  M  E  T  A  Q  T  E  V  A  Q  M  G  P  A  P  M  E  P  A  Q  M  E
28120 GTTGCCCAGGTAGAATCTGCTCCCATGCAGGTGGTCCAGAAGGAGCCTGTTCAGATGGAGCTGTCTCCTC
       V  A  Q  V  E  S  A  P  M  Q  V  V  Q  K  E  P  V  Q  M  E  L  S  P
28190 CCATGGAGGTGGTCCAGAAGGAGCCTGTTCAGATAGAGCTGTCTCCTCCCATGGAGGTGGTCCAGAAGGA
      P  M  E  V  V  Q  K  E  P  V  Q  I  E  L  S  P  P  M  E  V  V  Q  K  E
28260 ACCTGTTAAGATAGAGCTGTCTCCTCCCATAGAGGTGGTCCAGAAGGAGCCTGTTCAGATGGAGTTGTCT
        P  V  K  I  E  L  S  P  P  I  E  V  V  Q  K  E  P  V  Q  M  E  L  S
28330 CCTCCCATGGGGGTGGTTCAGAAGGAGCCTGCTCAGAGGGAGCCACCTCCTCCCAGAGAGCCTCCCCTTC
       P  P  M  G  V  V  Q  K  E  P  A  Q  R  E  P  P  P  P  R  E  P  P  L
28400 ACATGGAGCCAATTTCCAAAAAGCCTCCTCTCCGAAAAGATAAAAAGGAAAAGTCTAACATGCAGAGTGA
      H  M  E  P  I  S  K  K  P  P  L  R  K  D  K  K  E  K  S  N  M  Q  S  E
28470 AAGGGCACGGAAGGAGCAAGTCCTTATTGAAGTTGGCTTAGTGCCTGTTAAAGATAGCTGGCTTCTAAAG
        R  A  R  K  E  Q  V  L  I  E  V  G  L  V  P  V  K  D  S  W  L  L  K
28540 GAAAGTGTAAGCACAGAGGATCTCTCACCACCATCACCACCACTGCCAAAGGAAAATTTAAGAGAAGAGG
       E  S  V  S  T  E  D  L  S  P  P  S  P  P  L  P  K  E  N  L  R  E  E
28610 CATCAGGAGACCAAAAATTACTCAACACAGGTGAAGGAAATAAAGAAGCCCCTCTTCAGAAAGTAGGAGC
      A  S  G  D  Q  K  L  L  N  T  G  E  G  N  K  E  A  P  L  Q  K  V  G  A
28680 AGAAGAGGCAGATGAGAGCCTACCTGGTCTTGCTGCTAATATCAACGAATCTACCCATATTTCATCCTCT
        E  E  A  D  E  S  L  P  G  L  A  A  N  I  N  E  S  T  H  I  S  S  S
28750 GGACAAAACTTGAATACGCCAGAGGGTGAAACTTTAAATGGTAAACATCAGACTGACAGTATAGTTTGTG
       G  Q  N  L  N  T  P  E  G  E  T  L  N  G  K  H  Q  T  D  S  I  V  C
28820 AAATGAAAATGGACACTGATCAGAACACAAGAGAGAATCTCACTGGTATAAATTCAACAGTTGAAGAACC
      E  M  K  M  D  T  D  Q  N  T  R  E  N  L  T  G  I  N  S  T  V  E  E  P
28890 AGTTTCACCAATGCTTCCCCCTTCAGCAGTAGAAGAACGTGAAGCAGTGTCCAAAACTGCACTGGCATCA
        V  S  P  M  L  P  P  S  A  V  E  E  R  E  A  V  S  K  T  A  L  A  S
28960 CCTCCTGCTACAATGGCAGCAAATGAGTCTCAGGAAATTGATGAAGATGAAGGCATCCACAGCCATGAAG
       P  P  A  T  M  A  A  N  E  S  Q  E  I  D  E  D  E  G  I  H  S  H  E
29030 GAAGTGACCTAAGTGACAACATGTCAGAGGGTAGTGATGATTCTGGATTGCATGGGGCTCGGCCAGTTCC
      G  S  D  L  S  D  N  M  S  E  G  S  D  D  S  G  L  H  G  A  R  P  V  P
29100 ACAAGAATCTAGCAGAAAAAATGCAAAGGAAGCCTTGGCAGTCAAAGCGGCTAAGGGAGATTTTGTTTGT
        Q  E  S  S  R  K  N  A  K  E  A  L  A  V  K  A  A  K  G  D  F  V  C
29170 ATCTTCTGTGATCGTTCTTTCAGAAAGGGAAAAGATTACAGCAAACACCTCAATCGCCATTTGGTTAATG
       I  F  C  D  R  S  F  R  K  G  K  D  Y  S  K  H  L  N  R  H  L  V  N
29240 TGTACTATCTTGAAGAAGCAGCTCAAGGGCAGGAGTAATGAAACTTTGAACAAGGTTTCAGTTCTTAGTT
      V  Y  Y  L  E  E  A  A  Q  G  Q  E  *
29310 TGTAAGGTATATTACATTTTATATTCATTTATGATAGCAGACAACCTTTTAAGATTGCTTTAATTAGTAT
29380 CTGATGTTGATTTTTAAGTGGCATTCTTTTCCTTAGGACTTTTTATGTATACCTGTTGATTGTTGTGTAA
29450 ATTTTAGTAAATCTAAGAGAGTGTACTAAACCAGCAGGTATCTGTTAGCTTATGTGTTTAATTGAAATTA
29520 GAAGGCTAAGATGGTATAACAGCATTTTATTGCTTTGTCCAGCTACAACTTGTCATTTTTTTCTCCATGT
29590 CTTATCTTCCTGTTTCACTTTAGTTTATTCTTCGTTTTTTATTGAGATCTATAAAAAATTGGCTTACTTA
29660 ATAGCAAATTACTTGAAGAATTTGCCTGCTTTATATAAAGTTAGCACTTTAAGATTTTTTTTTTTAGAGA
29730 TGAGAAGACATTTAAATTGAAGAAAAATTCCCCCAGCAATAGACAGTCTATCAGTCCAAGTATTTACTTC
29800 CTGAGTTTTGATCAATATTTTTTATTTGTGTATGTTAATCGTCATAAAAACAGTGATTTTGGTGTGTTTT
29870 TTATTTTGGTGCTTTAATGGCTTAAGATGTTGCACATTTTTTTTTTCTTTTGGTTTCTGTTTATGTTTTT
29940 TTGCCTATGCAGTTAAATTTTTCCTAGAAATAGCATTTGTGTTGAACAGTAACACTTTATACATATATAT
30010 ATGCATGTTTATTTTGTTTGGCGTCTTTGGAGGGATGCTTTTAGACTTGTTTGCAAAAGGGCAGTTTTCT
30080 TTTTCTTTGCTGCAGTTGTCTATTTTGCAGAATAATAGTGTGTGCAAGTTTGTGAGCAAATGAAATATGC
30150 AGGTTCAATCTATTGATTTTGATTTTTACATCTTATATCTATGCCAGAATCTGTATTTCATATAACTTAT
30220 TTATTTCGAATGGATGTAGTAAATTCACAGCTATCAGTTTTGATTTTGCAATAAATAAACCACTAGGTTG
30290 CATGTCGAACAAATTTTTATCTCAAATACCAACCATCAGTTTTTTTTTTCATGTGTTTTGGTACAGCTAA
30360 TTCCTAATTGTAGAGTGTTAAATGTTTGAGGAGAACCTTTTCTCATAGATGGTTGGTGTTCATATGGCTA
30430 CTTTACAATAAAGAGAACTGTAAGTGATATTTGGAAACTACAAACCTGGAATTAGGAGATATAATTATTC
30500 CTTCAAGTTTTATAGAATATCACTTGGGAGATTCCAAAGCCATAGCTATTACGCGGCAAACCTAGGATAA
30570 GAAAGGTAGTATGAGTGCTGGTAGACCAGCTGCAACTTTCCTATACAGTGAAAAAGGCTGGTGAAACAAG
30640 TACAGTCCAGATTTTTTAAAATCATACTTTCTCAGGGATCTCCACAAACTGGTGGGTGTCCTGGCTGTCT
30710 GTGTGATAGCCTCTTTCTATAGGTGAGGCCTCAAATGAATTGCAGCTATCCTGGTGTTCCTATGAGGGCA
30780 CTTTGTATGAAAAAGGGCATGTACTCCAAAACATTTTTGTAGGTTCTTTGGCCAGTTGCCAAAGAGTGTG
30850 AAAGAATCCAATAGAGGATTTTTCTTACTGATAGCAGTCATTCATTGCAGTAAAATAAAATATGATCCCA
30920 TTAGGGAATCTTGAATTCTGACCTCCCATACTCCGTTTTGAAATAACCACTTTATATTTCATTTTTTAAA
```

30990 AATCTGATGATCTCTTTGAGGCAGGTTTCAGATTTGGCAGTACAACATGAAAGATTAGGAAAAGCATTAA
31060 TAACGTGTGGGTGGAAAGCTTGTTAAAAATCTGAGAGTGAAGTTTGAGTTAAAAGTTGTTTGAACATGGC
31130 ATTGACTGGGAGGCCAAAGATTTAAAGAAGCGGAAGATTCTTCTCTTAAGACATGAGGAGTAAGTTGTGT
31200 GATAATGGTATGTGTTTTGTGTGCATGAATGGACATTGTAAATGTTGAATTCTAGGCTCCGACAATCATT
31270 GTCAACAGAAGATCAAGCTGCAAATATTTATGTTTTAAAACTTAAATTATAAAGCTAGTTAAGTCTTTCT
31340 AATGACTAGTTTTAATGTTCATGGGTACATTTTACCTAAGTTACCGTTTACATTGTATAGAAAAAGATAC
31410 ATCTTAAGCACAGATTGGTTATTAGGAATTAGTTTGGGGAAGAGGTTTTTTTGTGGATTCTTTCATACTG
31480 CAAAGAAAAACCATTTGCCTTTTGGGGAATTGAGCTAACTTCTAATCTAGTCTTAAGACTAGAATGCTAA
31550 AAACAAAAACATGAAGGAAATTAAAACCCCTTATTATTAAATTGATTTGTAAAAACATTGTTACTGGAAA
31620 TTTATTGGACTTGAGGCCTTCCTCCAGAAAATAAGGACTTGATTGTCAGGCCTATATTAGGTTCTGAACC
31690 TTAATGCCATGTATTTGTACTTACTAAAAATTGTTTCAATGAAAAGTACATTAGCAGTATGAACTTCTGG
31760 TCCAGTTGGAAGTTTTTCCATTTGAAAAATGTGATGTTTGCATGGAACTGTTTGAAACTTTTTTATTTTC
31830 TAGTCCCCCTCCCCCACACTGGATAGAATTTAGCCTAGAATTTTCCCTTTGGATAAAAGAACAAAAATTG
31900 AACATGTTATTTGTAAATTGATGTTTAGTAATTAGTGATAAACTTGAAATACTAGCATATATTATAAGCC
31970 TTAATCTTAGGTAGTCTTATGAAAATGAATCTCTTAACTATCTTTTGAACCTGTATTCACATTGGTTTTC
32040 AAGATATTTTAAGTTATATTTTTTCCTCTTTTCAGAGCTGCTTCTTATTCTGGGGCTACTTTTTTTTTTA
32110 GTTGTGTAATTCACAAAGGGCTGCATTTTTTTTTTTTTTTTAATAAGGCTTATAACTATGGCTGGATCTTT
32180 TGCTCTAGTCTTCTAAGAAGGGCCATTTTATTTTTTAGAGTCACTTCTAAAGTCATGTGGTAATTAACTT
32250 TGGAGACTGTTTTGCGTATGAGTGCTGATACAAATTAAAACCCAAGTAGACCTCATTGCATGTCACCCTA
32320 TGAATGTTGACAATGGAAGGAATACCTTGCCTGTAGTATACTGTCACTTCTGGATTGATAAGCTGAGGAA
32390 GAAAGTTAAGTTTCTTTTTTACATAAGTCAGAAAAACTTACAGCTGGTGTTCCTAGTTTCCTGGTTGACC
32460 TCAGCAGATGAAGTGAACAGATAGTGTTAATTCAGATTGAAGAAATTATCTGAATCTTGGTTTGTGTAGA
32530 TTTACAATCTACATGCAATATTAACTAAATCAGATAGCTTTTACAGTTTCACATGTGTACATAGGTTCCC
32600 TCCCGGTCCCTTCCATATCCATTAGTTATTGAACTTTCTAAACTGGCATTGAAACATTACAACAATGTTT
32670 TGTTGCACCAATTTTATAAACTTAAGCAGTGCAATACGTGTTACTTTTCTGAGGCAAACCAAAGGTAAAT
32740 TTCTCAAGGTTCTTGCTGCCTTCTTTAGCAGCATTTGATGGAAGATCTTTTATACATTTGTAATAGATAA
32810 AAATAAACCAGATTGCAAATCCTTTTTTAAAATCCTAAACCATGTACCAAGTTTTTGGTCCAAATTATGT
32880 AGGATAAGTTAAACTTAAATTGCATTCTATTAACCAATATGAGTGTATTTCTGTAAGCATAGTTATGTTG
32950 AAATAAAGTTTTAAAAACCATGATGCCTTTGTTTATTATTGGTATATCCAGAATGTAGGAAAGCCAAATT
33020 TATGTTGCTGGAACTGCAAGTGTATTAATAGGCTTTTGAAGATTATTTCTCAGGCTGGGCGCAGTGGCTC
33090 ACGCCTGTAATCCCAGCACTTTGGGAGGCTGAGGCAGGTGGATCATGAGGTCAGGATTTCAAGGCCAGCC
33160 TGGCCGACATAGTGAAACCACGTCTCTACTAAAAATACAAAAATTAGCTGCGTGTGGTGGCAGGCGCCTG
33230 TAATCCCAGCTACTCGGGAGGCTGAGGCAAGAGAATCGCTTGAACACAGGAGGTGGAGGTTGCAGTGAGC
33300 CGAGATCGTGCCATTGCACTTCAGCCCAGGCGAGAGTGTGAGACTCTGTCTTTAAAAAAAAAAAAAAAAA
33370 AAGTATCACAGGTAGAAAAGTCACAGCATGTTGTCTGAGGTAACAGTCCAGTATGGGTAGTAACCCAAAG
33440 CATACACTGCTTGATGGGAGGTGCGGGAGAAATTGGACAATTCACACAAGATTTTAAATAGTAATTACTT
33510 CCCAACCACAAGTCCCCTTAATAGTTTTCATATGCATATCCCTTTTCAGTAGCAGGCCTGGAATTAGTGA
33580 ATTTTGATTCATGAAGATGTTTTATTTTATGTATTTATTTTTTTTTGAAACAGTCTCTGTCGCCCAGGCT
33650 GGAGTGCAGTGTCATGATCCTAGCTCACTGCAACCTCTGCTGCCCGGGTTCAAGCAACTCTCCTGCCCCA
33720 GCCTCGCAAGTAGCTGGGATCACAGGCGCCCACCACCGCGCCTGGCCAATTTTTGTATTTCTAGTAGAGA
33790 CGGGGTTTCACCACGTTGGCCAGGATGGTATCGATCTCCTGACCTCCTGATCCACCCGCCTCAGCCTCCC
33860 AAAGTGCTGGGATTACAGGCATGAGCTACCGTGCCCGGCTGTTTTTTATAAAATGAGACGGAGTTCTGTG
33930 TTGCCCAGGGTGGACTCAGATGATCCTCCCACCTCAGTCTCCCTAAATGCTGGGATTACAGATGTGAGCC
34000 ACCATGCCTGGCCTGTGTGTTTTTTTTTTTTTTCTTCTCCCAGACGGAGTCTTGCTCTGTCGCCCAGACTG
34070 GAGTGCCATGTCATGATCTCCTTTCACTGCAACCTCGGCCTCCAGGTTCAAGCGATTCTCCTGCCTCAGC
34140 CTCCTGAGTAACTGGGATTACAGGTGTCCGCCACAACACCCAGCTAATTTTTGTATTTTAGTAGAGATGG
34210 GGTTTCACCATGTGGCCCAGGCTGGTCTCGAACTCCTGAGCTGAAGTATTCCTCCGACTTTGGCCTCCCA
34280 AAGTGCTGGGATTACAGGTGTGAGCCACCACGCCCAGCCTGTTGTTTTTTTGTTTTGAGAAACGGTCTTA
34350 CTCTGTCTCCCAGGCTGGAGTGTGGTGGCATGATCACAGTTCCTGCAGCCTTGAACTCCTGGGCTCAAGT
34420 GGTCTTCTTGCCTCAGCCTCCTGAGTAGCTGGGACTACAGACCTACCCCACCACGCCTCTTGCCGCCTCC
34490 TCTCAAAAGCGTTCGTATTATAGGCATGAGCCATCATGCCTTGCCACATTTTGTTTTGATGCAGTGTCAG
34560 ATCTGGAAGATTCTGAGTCAGGAGAATGGTATTTCATATTTGGATTGGTGAGTTATTCACCCTCTTCCCA
34630 CTTCCTGCAAAACTCCTTATCAGCAACAAAGCAAAATTAACTTTTTTTTCCTCTGAGACAGGGTCTTGCT
34700 CTGTTGTCCGGGCCGTAGTGCAGTGGCACCATCATGGCTCACTTCTGCCTCAACCTTCTGGACTCAAGCA
34770 AACCTCCCACCTCAGCCTCTTGGAGTAGCTGGAACTACAGGTGCATGCCACACACCCAGCTAGTTTTTTT
34840 CTTTCTTCTTTTTTTTCTATTGACAGGGTTTTGCCTTGCTCCCCAGGATGGTCTTGAACTCTTGGACTCA
34910 GGCAGTTCACCTGCTTCGGCCTCCTAAAGTGCTGGGATTACAGGCGTGAGCCACAATGCC
