# Supplementary material for: Neuro293: A REST-knockout HEK-293 cell line enables the expression of neuron-restricted genes for the high-throughput testing of human neurobiology and the biochemistry of neuronal proteins
Source: Biol Methods Protoc. 2025 May 10;10(1):bpaf036. doi: 10.1093/biomethods/bpaf036 (PMC12202048; doi:10.1093/biomethods/bpaf036)
Supplement: bpaf036_Supplementary_Data [file bpaf036_supplementary_data.zip › Supplemental Materials 3 RNA Sequencing.zip!/Supplemental Materials 3 RNA Sequencing/Supplementary Materials 3 Bioinformatics Plots.zip!/RESTKOvsWT.all_DOdot.pdf]

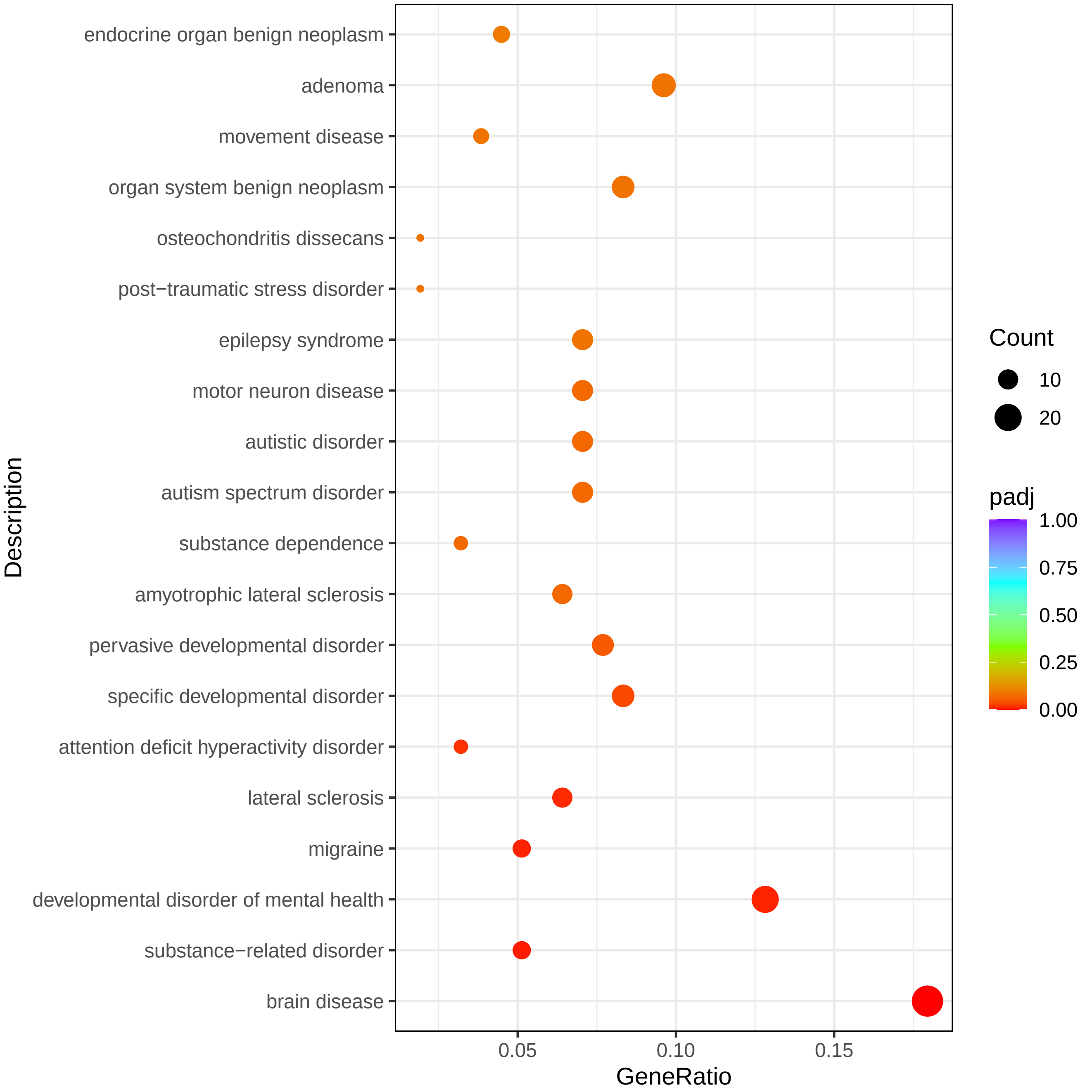

endocrine organ benign neoplasm
adenoma
movement disease
organ system benign neoplasm
osteochondritis dissecans
post−traumatic stress disorder
epilepsy syndrome
motor neuron disease
autistic disorder
autism spectrum disorder
substance dependence
amyotrophic lateral sclerosis
pervasive developmental disorder
specific developmental disorder
attention deficit hyperactivity disorder
lateral sclerosis
migraine
developmental disorder of mental health
substance−related disorder
brain disease
Description
0.05
0.10
0.15
GeneRatio
Count
10
20
padj
1.00
0.75
0.50
0.25
0.00
