# Supplementary material for: Neuro293: A REST-knockout HEK-293 cell line enables the expression of neuron-restricted genes for the high-throughput testing of human neurobiology and the biochemistry of neuronal proteins
Source: Biol Methods Protoc. 2025 May 10;10(1):bpaf036. doi: 10.1093/biomethods/bpaf036 (PMC12202048; doi:10.1093/biomethods/bpaf036)
Supplement: bpaf036_Supplementary_Data [file bpaf036_supplementary_data.zip › Supplemental Materials 1 REST KO sequence and Western Blots.zip!/Supplemental Materials 1 REST KO sequence and Western Blots/Supplemental Materials 4 Raw Western Blots.pdf]

1st Incubation
800 Channel
Synapsin 1

WT KO WT KO WT KO

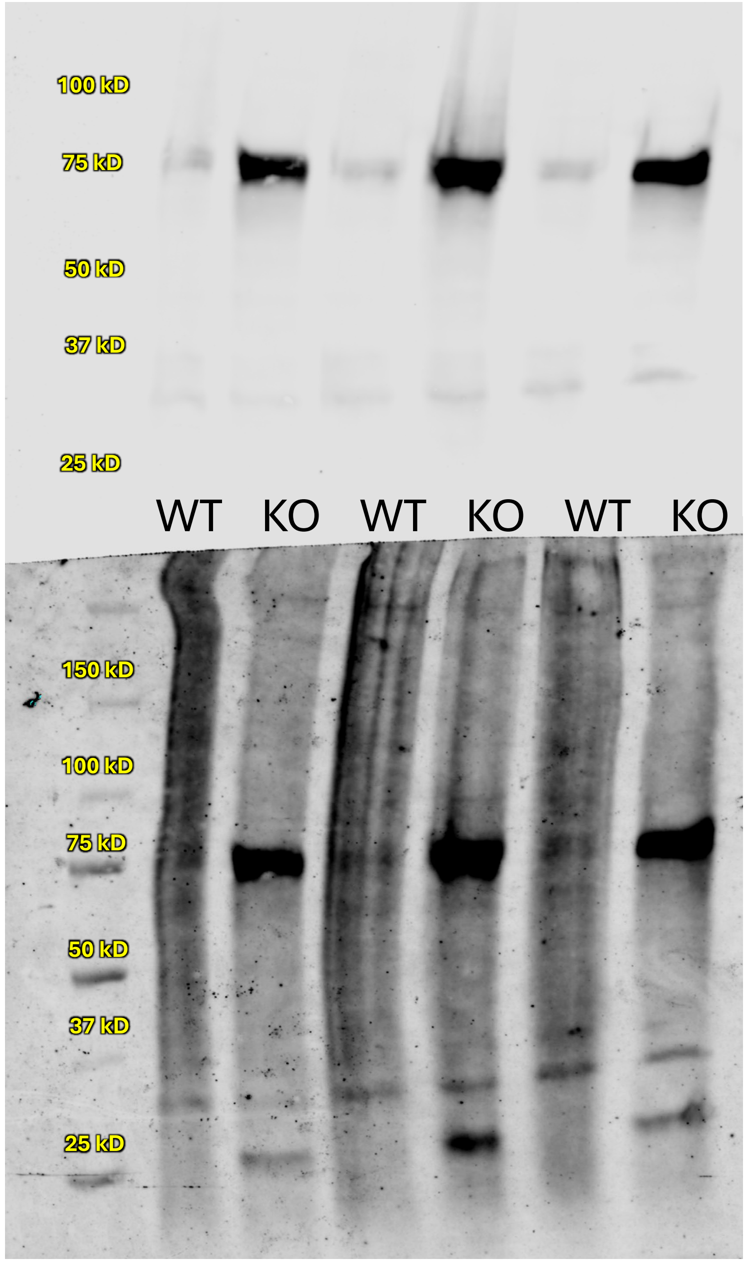

Synapsin 1
~75 kD

2nd Incubation
800 Channel
Add Snap25

Synapsin 1

Snap25
~25 kD

1st Incubation
700 Channel
Kv1.2

WT KO WT KO WT KO

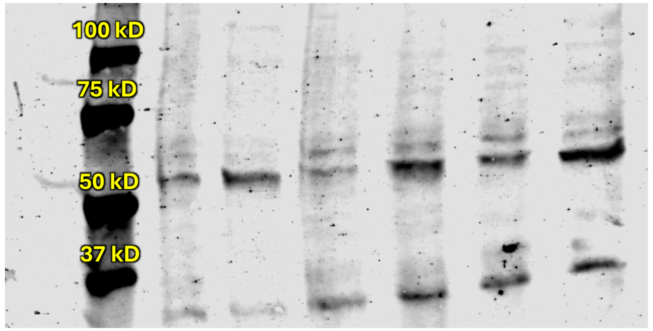

Kv1.2
~ 62 kD

2nd Incubation
700 Channel
Add Beta-Actin

WT KO WT KO WT KO

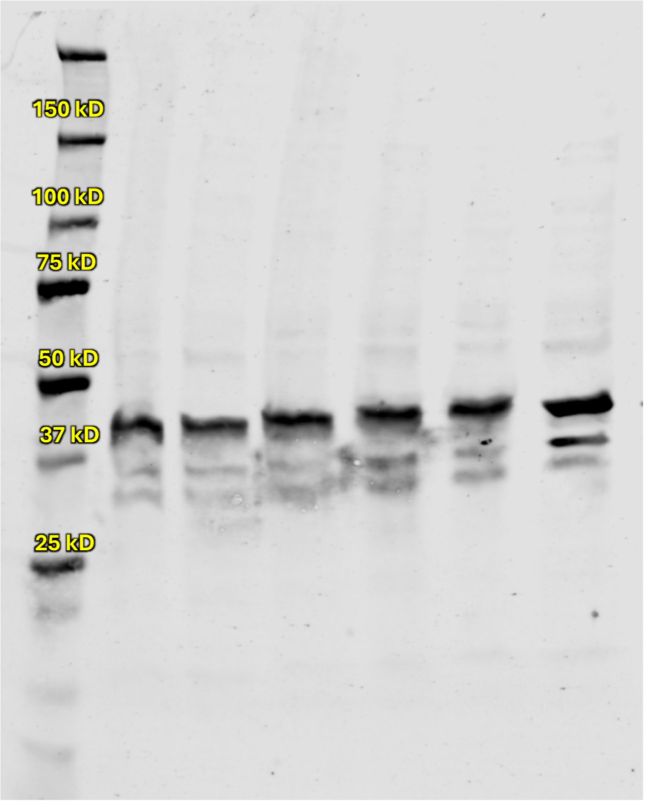

Beta-Actin
~42 kD

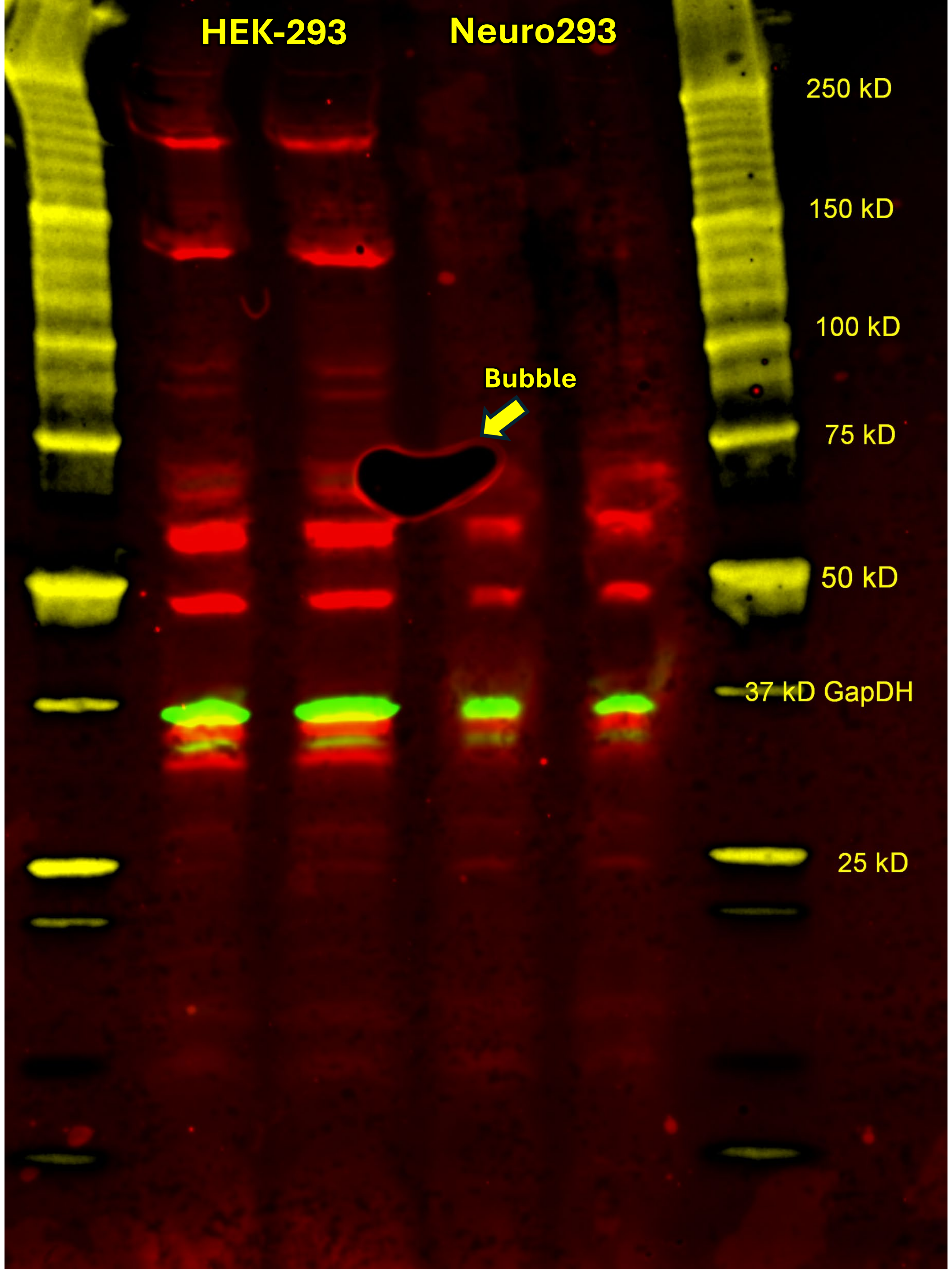

REST
~200 kD

GapDH
~37 kD
(Green)
