# Supplementary material for: Neuro293: A REST-knockout HEK-293 cell line enables the expression of neuron-restricted genes for the high-throughput testing of human neurobiology and the biochemistry of neuronal proteins
Source: Biol Methods Protoc. 2025 May 10;10(1):bpaf036. doi: 10.1093/biomethods/bpaf036 (PMC12202048; doi:10.1093/biomethods/bpaf036)
Supplement: bpaf036_Supplementary_Data [file bpaf036_supplementary_data.zip › Supplemental Materials 3 RNA Sequencing.zip!/Supplemental Materials 3 RNA Sequencing/Supplementary Materials 3 Bioinformatics Plots.zip!/RESTKOvsWT.all_GObar.pdf]

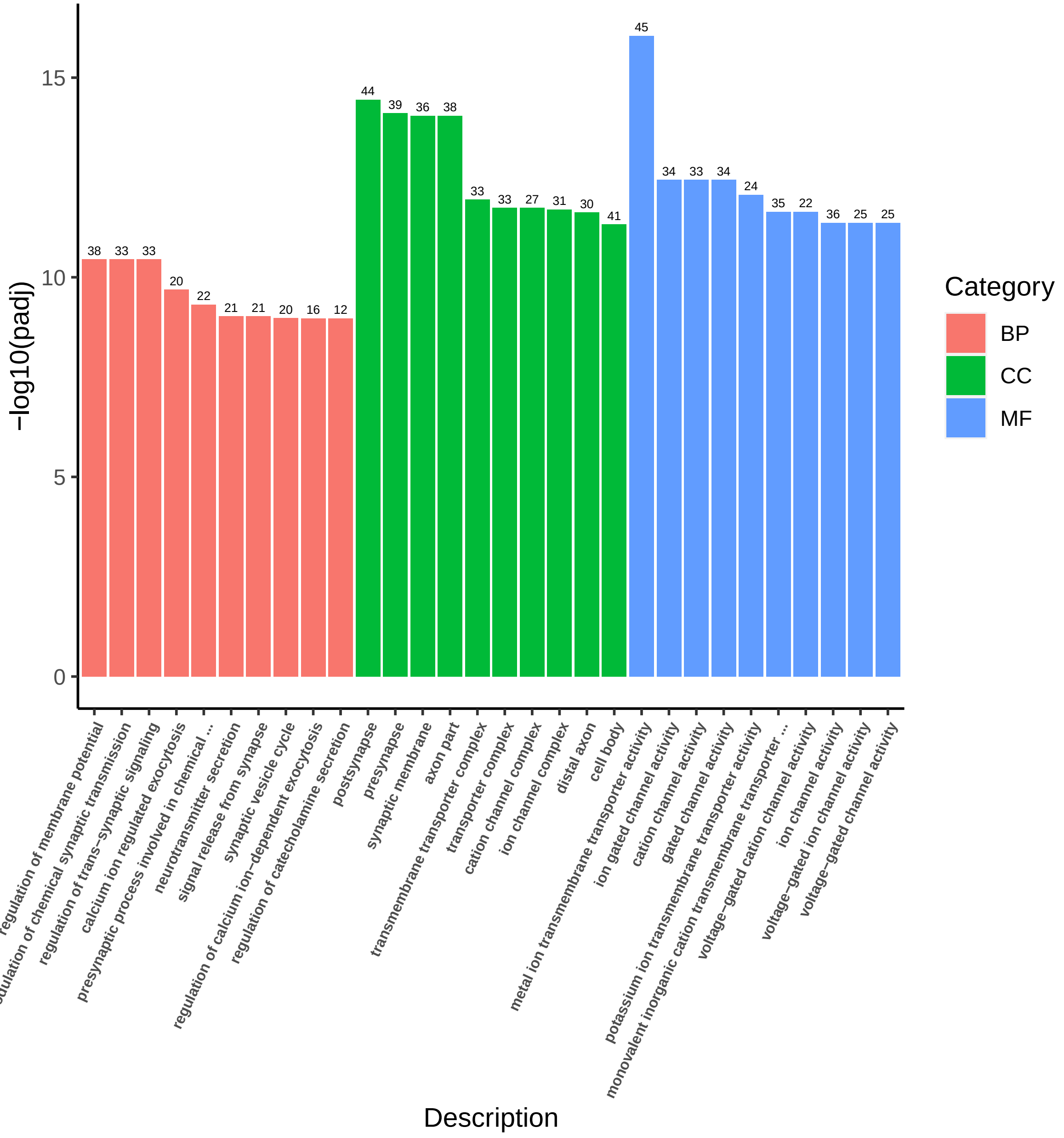

−log10(padj)
15
10
5
0
38
33
33
20
22
21
21
20
16
12
44
39
36
38
33
33
27
31
30
41
45
34
33
34
24
35
22
36
25
25
Category
BP
CC
MF
regulation of membrane potential
modulation of chemical synaptic transmission
regulation of trans−synaptic signaling
calcium ion regulated exocytosis
presynaptic process involved in chemical ...
neurotransmitter secretion
signal release from synapse
synaptic vesicle cycle
regulation of calcium ion−dependent exocytosis
regulation of catecholamine secretion
postsynapse
presynapse
synaptic membrane
axon part
transmembrane transporter complex
transporter complex
cation channel complex
ion channel complex
distal axon
cell body
metal ion transmembrane transporter activity
ion gated channel activity
cation channel activity
gated channel activity
potassium ion transmembrane transporter activity
monovalent inorganic cation transmembrane transporter ...
voltage−gated cation channel activity
ion channel activity
voltage−gated ion channel activity
voltage−gated channel activity
Description
