# Supplementary material for: Neuro293: A REST-knockout HEK-293 cell line enables the expression of neuron-restricted genes for the high-throughput testing of human neurobiology and the biochemistry of neuronal proteins
Source: Biol Methods Protoc. 2025 May 10;10(1):bpaf036. doi: 10.1093/biomethods/bpaf036 (PMC12202048; doi:10.1093/biomethods/bpaf036)
Supplement: bpaf036_Supplementary_Data [file bpaf036_supplementary_data.zip › Supplemental Materials 3 RNA Sequencing.zip!/Supplemental Materials 3 RNA Sequencing/Supplementary Materials 3 Bioinformatics Plots.zip!/RESTKOvsWT.all_GObar_cc.pdf]

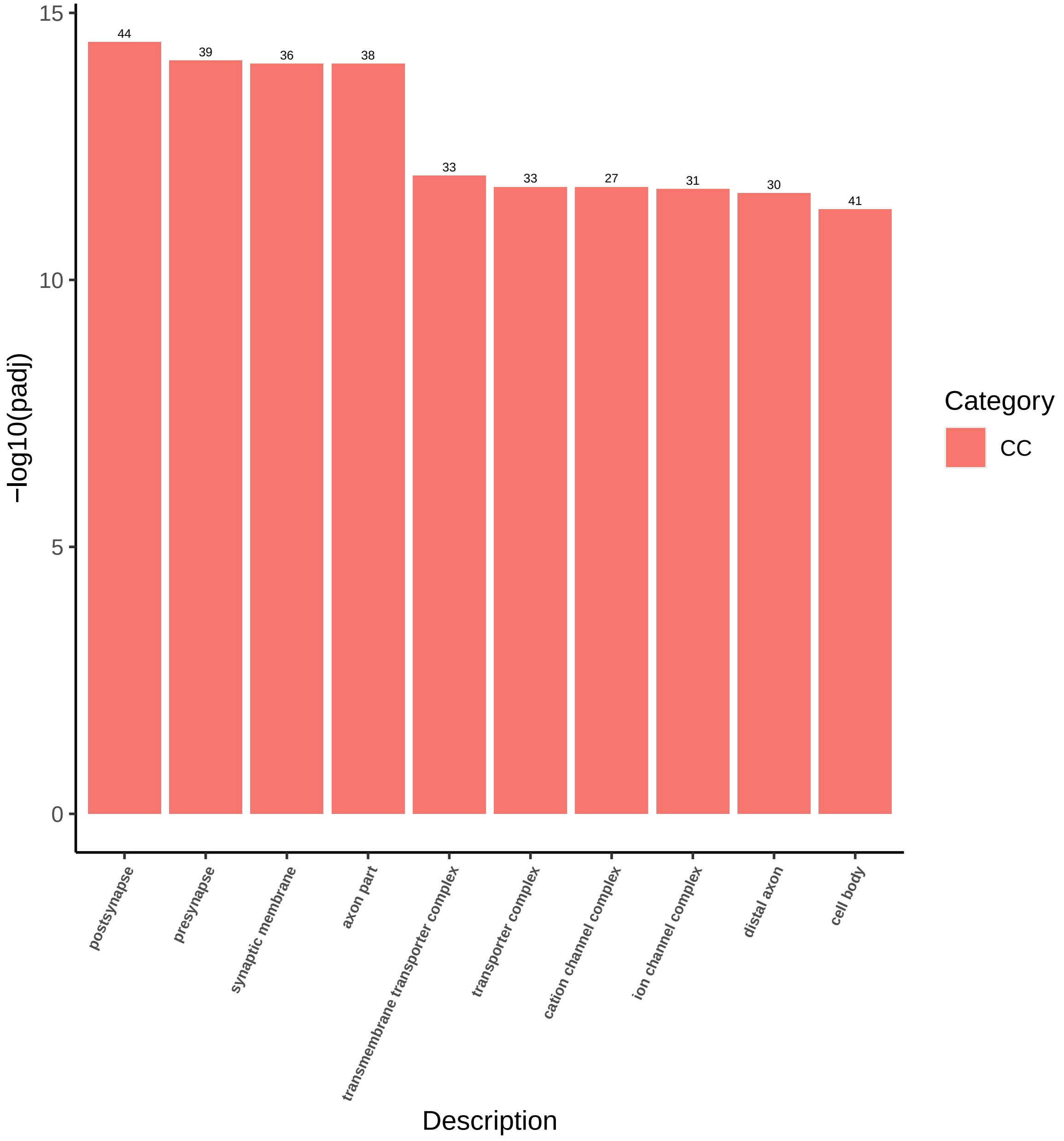

15
10
5
0
−log10(padj)
44
39
36
38
33
33
27
31
30
41
postsynapse
presynapse
synaptic membrane
axon part
transmembrane transporter complex
transporter complex
cation channel complex
ion channel complex
distal axon
cell body
Description
Category
CC
