# Supplementary material for: Neuro293: A REST-knockout HEK-293 cell line enables the expression of neuron-restricted genes for the high-throughput testing of human neurobiology and the biochemistry of neuronal proteins
Source: Biol Methods Protoc. 2025 May 10;10(1):bpaf036. doi: 10.1093/biomethods/bpaf036 (PMC12202048; doi:10.1093/biomethods/bpaf036)
Supplement: bpaf036_Supplementary_Data [file bpaf036_supplementary_data.zip › Supplemental Materials 3 RNA Sequencing.zip!/Supplemental Materials 3 RNA Sequencing/Supplementary Materials 3 Bioinformatics Plots.zip!/RESTKOvsWT.all_GObar_graph_updown.pdf]

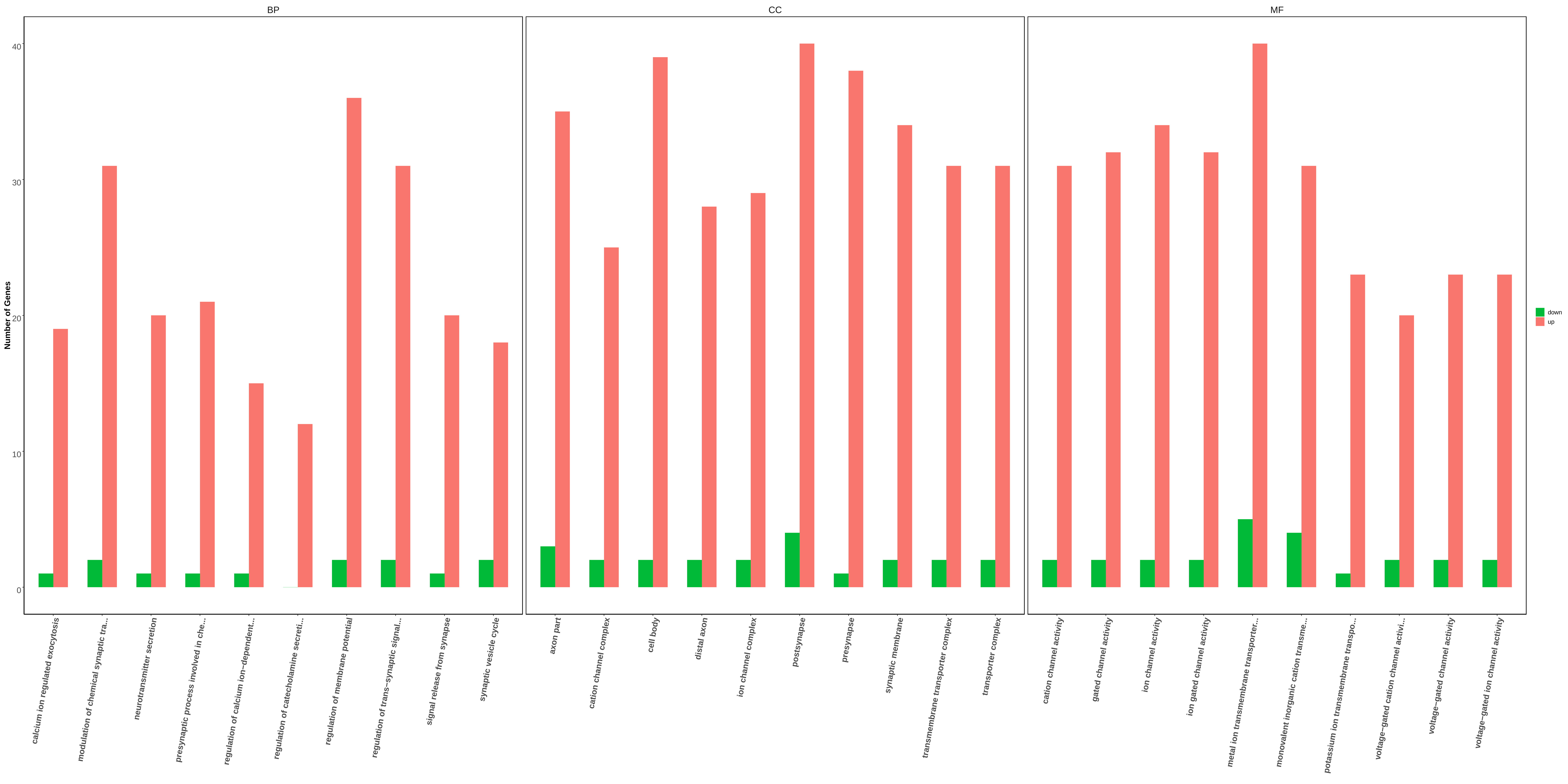
BP
CC
MF
Number of Genes
0
10
20
30
40
down
up
calcium ion regulated exocytosis
modulation of chemical synaptic tra...
neurotransmitter secretion
presynaptic process involved in che...
regulation of calcium ion−dependent...
regulation of catecholamine secreti...
regulation of membrane potential
regulation of trans−synaptic signal...
signal release from synapse
synaptic vesicle cycle
axon part
cation channel complex
cell body
distal axon
ion channel complex
postsynapse
presynapse
synaptic membrane
transmembrane transporter complex
transporter complex
cation channel activity
gated channel activity
ion channel activity
ion gated channel activity
metal ion transmembrane transporter...
monovalent inorganic cation transme...
potassium ion transmembrane transpo...
voltage−gated cation channel activi...
voltage−gated channel activity
voltage−gated ion channel activity
