# Supplementary material for: Neuro293: A REST-knockout HEK-293 cell line enables the expression of neuron-restricted genes for the high-throughput testing of human neurobiology and the biochemistry of neuronal proteins
Source: Biol Methods Protoc. 2025 May 10;10(1):bpaf036. doi: 10.1093/biomethods/bpaf036 (PMC12202048; doi:10.1093/biomethods/bpaf036)
Supplement: bpaf036_Supplementary_Data [file bpaf036_supplementary_data.zip › Supplemental Materials 3 RNA Sequencing.zip!/Supplemental Materials 3 RNA Sequencing/Supplementary Materials 3 Bioinformatics Plots.zip!/RESTKOvsWT.all_GObar_mf.pdf]

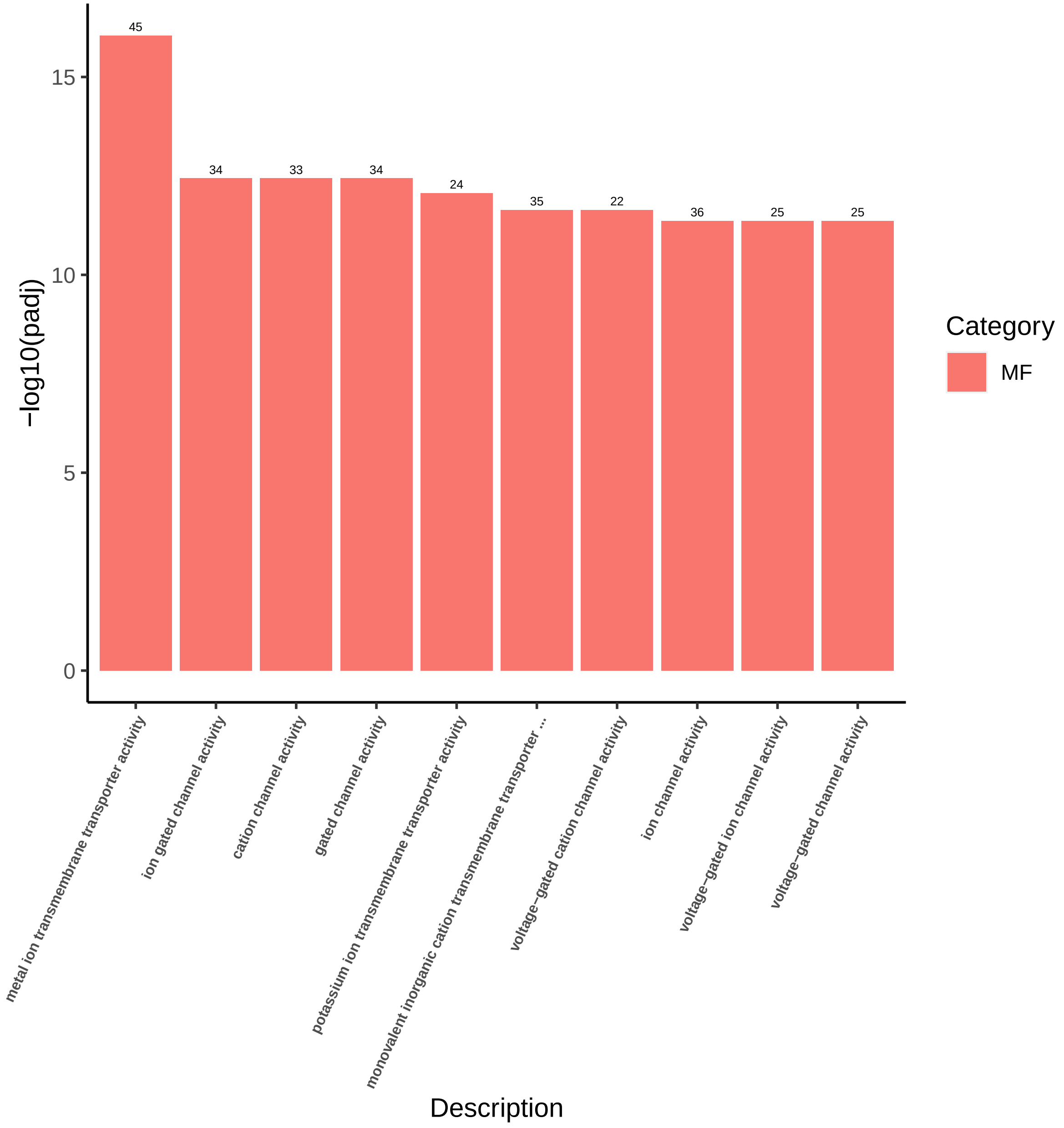

45
34
33
34
24
35
22
36
25
25
15
10
5
0
−log10(padj)
Category
MF
metal ion transmembrane transporter activity
ion gated channel activity
cation channel activity
gated channel activity
potassium ion transmembrane transporter activity
monovalent inorganic cation transmembrane transporter ...
voltage−gated cation channel activity
ion channel activity
voltage−gated ion channel activity
voltage−gated channel activity
Description
