# Supplementary material for: Neuro293: A REST-knockout HEK-293 cell line enables the expression of neuron-restricted genes for the high-throughput testing of human neurobiology and the biochemistry of neuronal proteins
Source: Biol Methods Protoc. 2025 May 10;10(1):bpaf036. doi: 10.1093/biomethods/bpaf036 (PMC12202048; doi:10.1093/biomethods/bpaf036)
Supplement: bpaf036_Supplementary_Data [file bpaf036_supplementary_data.zip › Supplemental Materials 3 RNA Sequencing.zip!/Supplemental Materials 3 RNA Sequencing/Supplementary Materials 3 Bioinformatics Plots.zip!/RESTKOvsWT.all_GOdot.pdf]

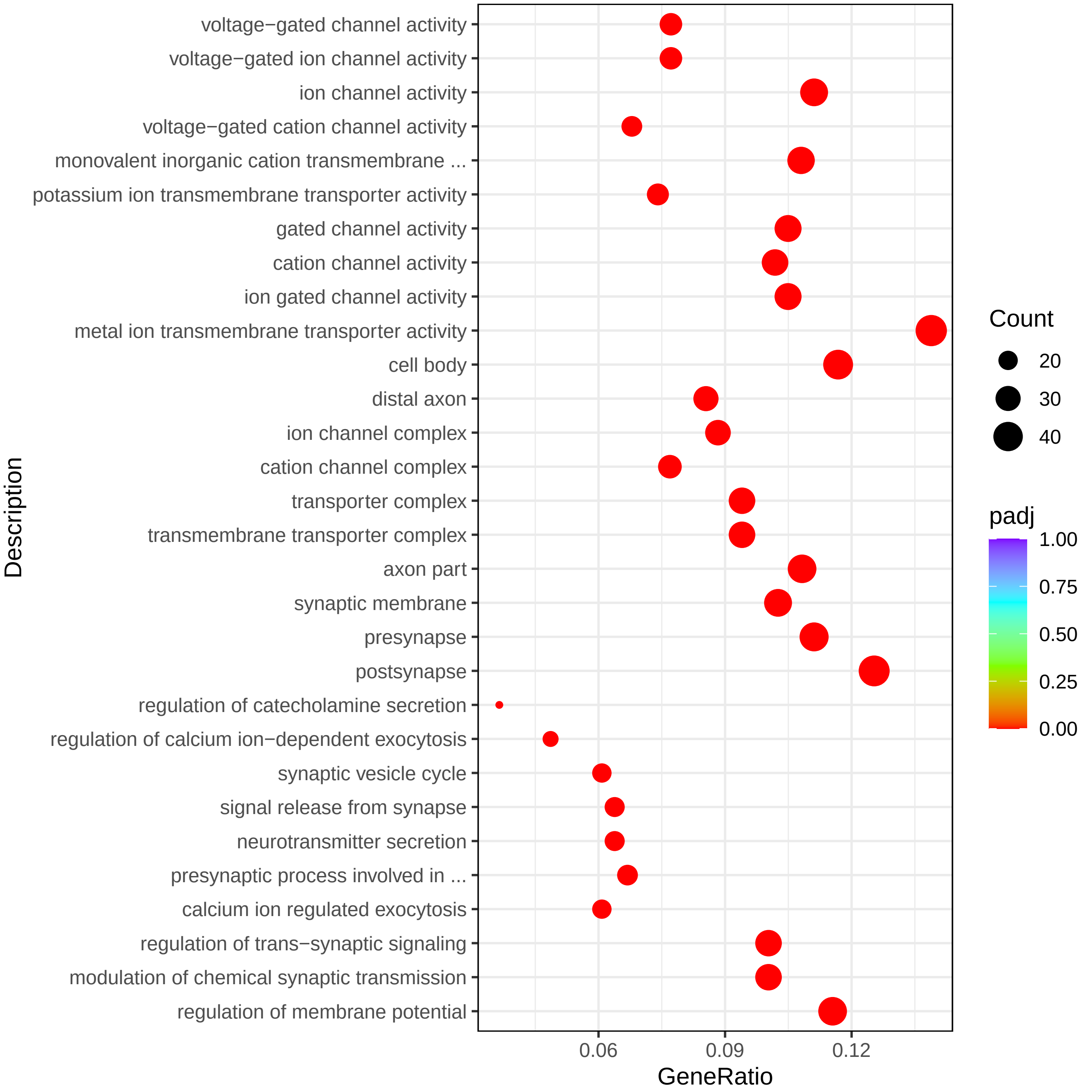
voltage−gated channel activity
voltage−gated ion channel activity
ion channel activity
voltage−gated cation channel activity
monovalent inorganic cation transmembrane ...
potassium ion transmembrane transporter activity
gated channel activity
cation channel activity
ion gated channel activity
metal ion transmembrane transporter activity
cell body
distal axon
ion channel complex
cation channel complex
transporter complex
transmembrane transporter complex
axon part
synaptic membrane
presynapse
postsynapse
regulation of catecholamine secretion
regulation of calcium ion−dependent exocytosis
synaptic vesicle cycle
signal release from synapse
neurotransmitter secretion
presynaptic process involved in ...
calcium ion regulated exocytosis
regulation of trans−synaptic signaling
modulation of chemical synaptic transmission
regulation of membrane potential
Description
0.06
0.09
0.12
GeneRatio
Count
20
30
40
padj
1.00
0.75
0.50
0.25
0.00
