# Supplementary material for: Neuro293: A REST-knockout HEK-293 cell line enables the expression of neuron-restricted genes for the high-throughput testing of human neurobiology and the biochemistry of neuronal proteins
Source: Biol Methods Protoc. 2025 May 10;10(1):bpaf036. doi: 10.1093/biomethods/bpaf036 (PMC12202048; doi:10.1093/biomethods/bpaf036)
Supplement: bpaf036_Supplementary_Data [file bpaf036_supplementary_data.zip › Supplemental Materials 3 RNA Sequencing.zip!/Supplemental Materials 3 RNA Sequencing/Supplementary Materials 3 Bioinformatics Plots.zip!/RESTKOvsWT.all_KEGGbar.pdf]

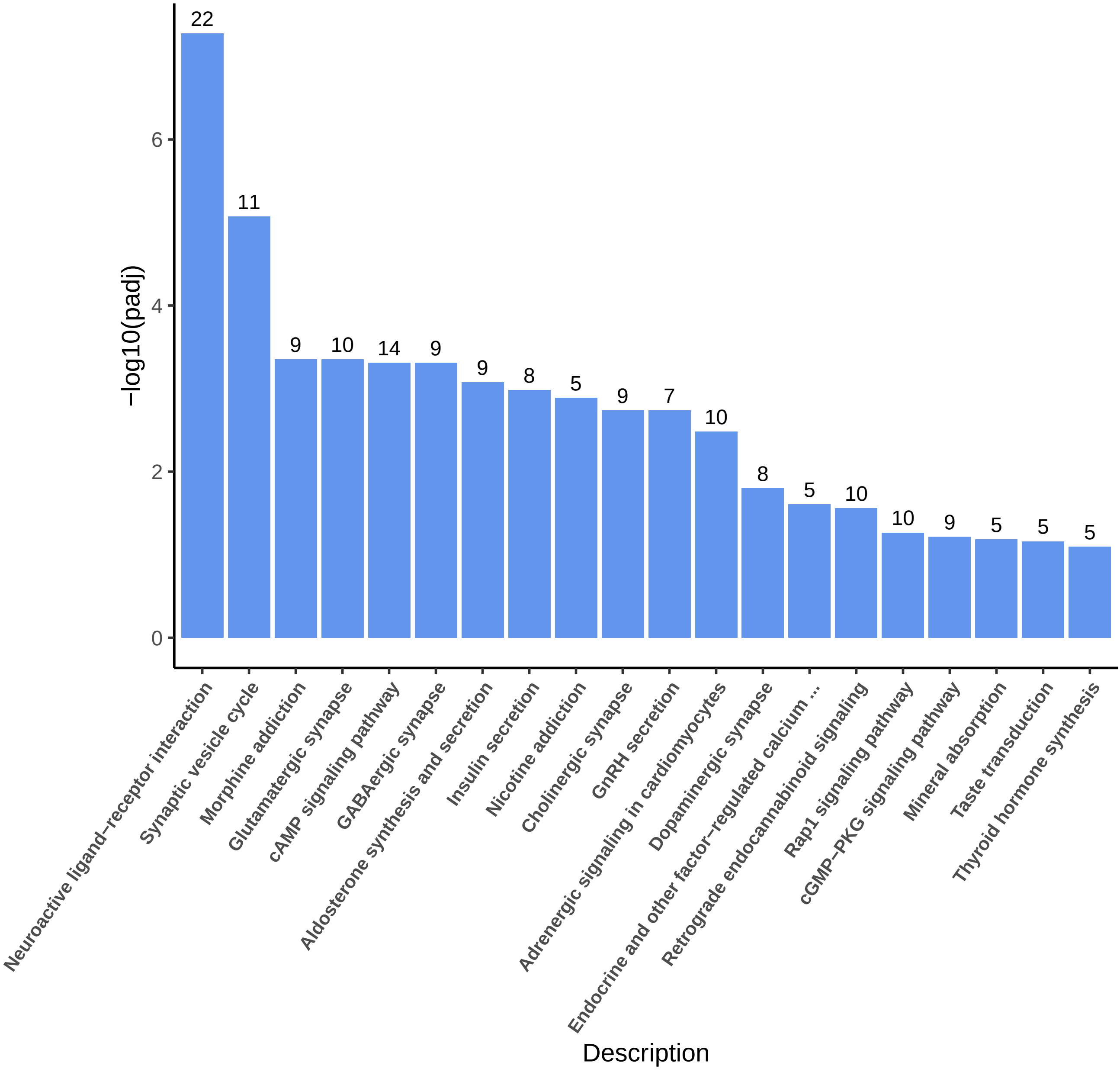

22
11
9
10
14
9
9
8
5
9
7
10
8
5
10
10
9
5
5
5
6
4
2
0
−log10(padj)
Neuroactive ligand−receptor interaction
Synaptic vesicle cycle
Morphine addiction
Glutamatergic synapse
cAMP signaling pathway
GABAergic synapse
Aldosterone synthesis and secretion
Insulin secretion
Nicotine addiction
Cholinergic synapse
GnRH secretion
Adrenergic signaling in cardiomyocytes
Dopaminergic synapse
Endocrine and other factor−regulated calcium ...
Retrograde endocannabinoid signaling
Rap1 signaling pathway
cGMP−PKG signaling pathway
Mineral absorption
Taste transduction
Thyroid hormone synthesis
Description
