# Supplementary material for: Neuro293: A REST-knockout HEK-293 cell line enables the expression of neuron-restricted genes for the high-throughput testing of human neurobiology and the biochemistry of neuronal proteins
Source: Biol Methods Protoc. 2025 May 10;10(1):bpaf036. doi: 10.1093/biomethods/bpaf036 (PMC12202048; doi:10.1093/biomethods/bpaf036)
Supplement: bpaf036_Supplementary_Data [file bpaf036_supplementary_data.zip › Supplemental Materials 3 RNA Sequencing.zip!/Supplemental Materials 3 RNA Sequencing/Supplementary Materials 3 Bioinformatics Plots.zip!/RESTKOvsWT.all_KEGGdot.pdf]

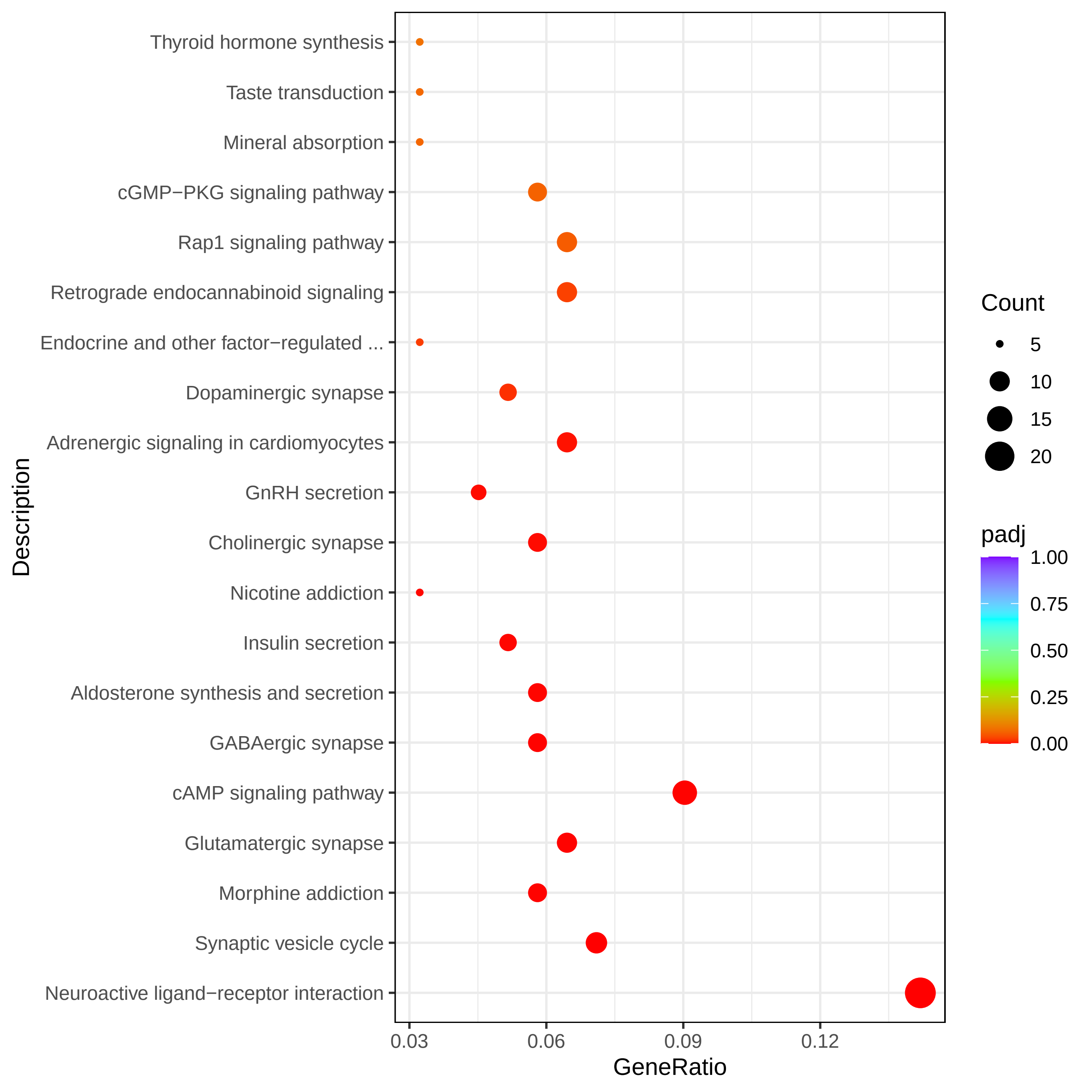
Thyroid hormone synthesis
Taste transduction
Mineral absorption
cGMP−PKG signaling pathway
Rap1 signaling pathway
Retrograde endocannabinoid signaling
Endocrine and other factor−regulated ...
Dopaminergic synapse
Adrenergic signaling in cardiomyocytes
GnRH secretion
Cholinergic synapse
Nicotine addiction
Insulin secretion
Aldosterone synthesis and secretion
GABAergic synapse
cAMP signaling pathway
Glutamatergic synapse
Morphine addiction
Synaptic vesicle cycle
Neuroactive ligand−receptor interaction
Description
0.03
0.06
0.09
0.12
GeneRatio
Count
5
10
15
20
padj
1.00
0.75
0.50
0.25
0.00
