# Supplementary material for: Neuro293: A REST-knockout HEK-293 cell line enables the expression of neuron-restricted genes for the high-throughput testing of human neurobiology and the biochemistry of neuronal proteins
Source: Biol Methods Protoc. 2025 May 10;10(1):bpaf036. doi: 10.1093/biomethods/bpaf036 (PMC12202048; doi:10.1093/biomethods/bpaf036)
Supplement: bpaf036_Supplementary_Data [file bpaf036_supplementary_data.zip › Supplemental Materials 3 RNA Sequencing.zip!/Supplemental Materials 3 RNA Sequencing/Supplementary Materials 3 Bioinformatics Plots.zip!/RESTKOvsWT.all_Reactomedot.pdf]

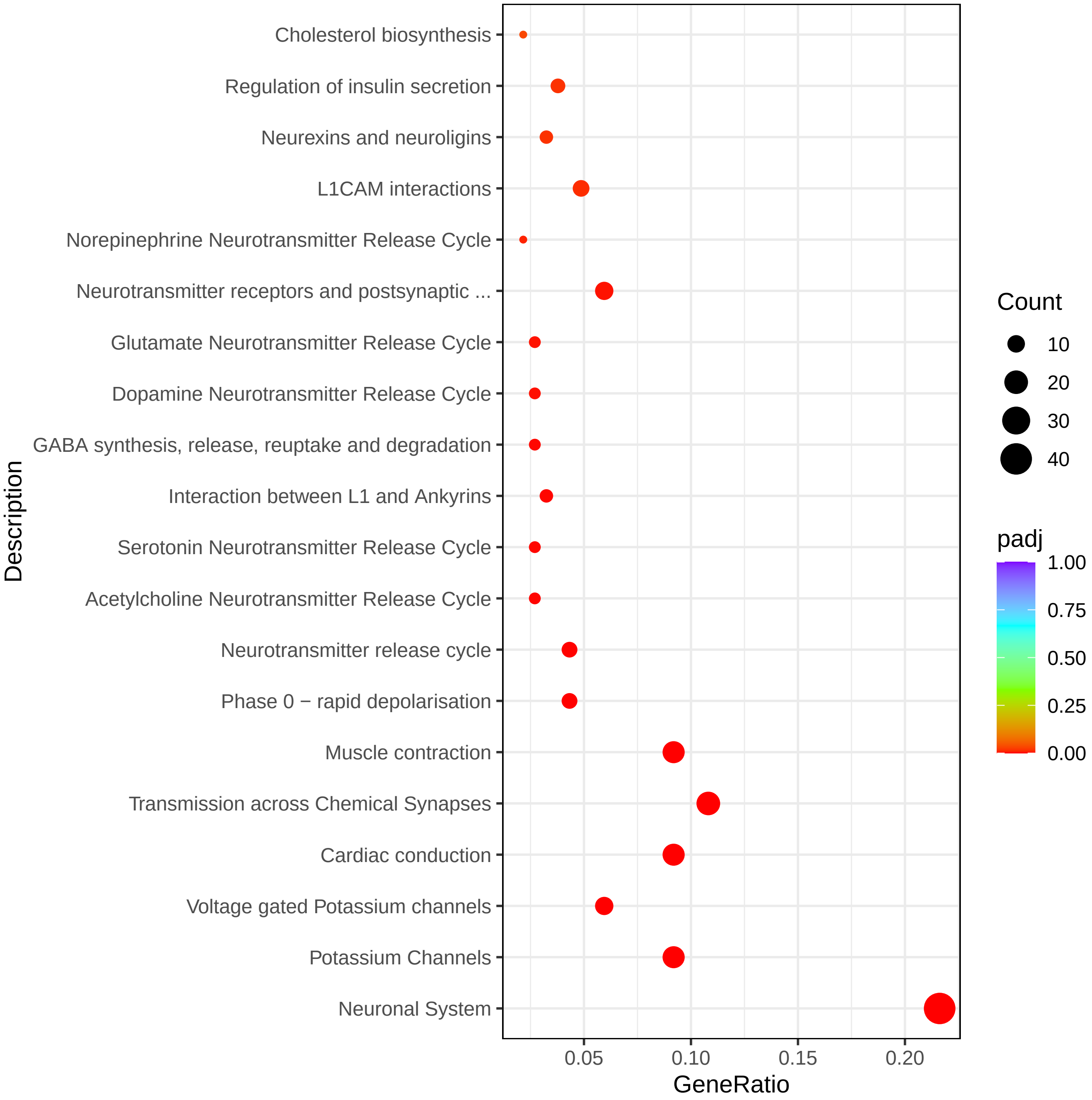

Cholesterol biosynthesis
Regulation of insulin secretion
Neurexins and neuroligins
L1CAM interactions
Norepinephrine Neurotransmitter Release Cycle
Neurotransmitter receptors and postsynaptic ...
Glutamate Neurotransmitter Release Cycle
Dopamine Neurotransmitter Release Cycle
GABA synthesis, release, reuptake and degradation
Interaction between L1 and Ankyrins
Serotonin Neurotransmitter Release Cycle
Acetylcholine Neurotransmitter Release Cycle
Neurotransmitter release cycle
Phase 0 − rapid depolarisation
Muscle contraction
Transmission across Chemical Synapses
Cardiac conduction
Voltage gated Potassium channels
Potassium Channels
Neuronal System
Description
0.05
0.10
0.15
0.20
GeneRatio
Count
10
20
30
40
padj
1.00
0.75
0.50
0.25
0.00
